# FTO-Driven m6A Demethylation of TNC Promotes Epithelial–Mesenchymal Transition and Gastric Cancer Metastasis

**DOI:** 10.3390/biomedicines14091958

**Published:** 2026-08-31

**Authors:** Jiao Deng, Shuang Liu, Feng Xia, Haokun Zhang, Zhen Sun

**Affiliations:** 1 GI Cancer Research Institute, Tongji Hospital, Tongji Medical College, Huazhong University of Science and Technology, Wuhan 430030, China; 2 Department of Gastrointestinal Surgery, Tongji Hospital, Tongji Medical College, Huazhong University of Science and Technology, Wuhan 430030, China; 3 Department of Gastrointestinal Surgery, The Second Affiliated Hospital of Kunming Medical University, Kunming 650101, China; 4 Department of Gastrointestinal Surgery, Peking University Shenzhen Hospital, Shenzhen 518036, China; 5 Department of Hepatic Surgery, Tongji Hospital, Tongji Medical College, Huazhong University of Science and Technology, Wuhan 430030, China; 6 Department of Surgery and Molecular Medicine Center, Tongji Hospital, Tongji Medical College, Huazhong University of Science and Technology, 1095 Jiefang Avenue, Wuhan 430030, China

**Keywords:** *FTO*, m6A methylation, tenascin-C, epithelial–mesenchymal transition, metastasis

## Abstract

**Background/Objectives**: Gastric cancer (GC) remains a leading cause of cancer-related mortality, with metastasis being the primary driver of poor prognosis. The extracellular matrix glycoprotein tenascin-C (TNC) is implicated in tumor progression and metastasis, yet its regulatory mechanisms in GC remain poorly understood. Here, we identify RNA N6-methyladenosine (m6A) demethylase fat mass and obesity-associated protein (FTO) as a key modulator of *TNC* expression, promoting epithelial–mesenchymal transition (EMT) and GC metastasis. **Methods**: Integrating The Cancer Genome Atlas Stomach Adenocarcinoma (TCGA-STAD) and Gene Expression Omnibus (GEO) datasets (GSE246567, GSE181840, GSE163126, GSM4837878), we demonstrate that TNC is significantly upregulated in GC and correlates with worse clinical outcomes. Functional assays both in vitro and in vivo reveal that *TNC* knockdown suppresses GC cell proliferation, migration, invasion, and tumor metastasis, while overexpression of *TNC* rescues these effects. Mechanistically, FTO selectively demethylates *TNC* mRNA, reducing m6A modification at the 3’ untranslated region, thereby enhancing *TNC* mRNA stability and expression. RNA pull-down assays identify YTH domain family protein 2 (YTHDF2) as the m6A reader that recognizes the methylated site on *TNC* 3’UTR, and *YTHDF2* knockdown partially rescues TNC expression and stability upon *FTO* loss. m6A RNA immunoprecipitation (MeRIP) and dual-luciferase reporter assays confirm FTO’s direct regulation of *TNC* via m6A demethylation. Furthermore, *TNC* promotes EMT by activating the phosphoinositide 3-kinase (PI3K)-AKT (protein kinase B) signaling pathway, as demonstrated by rescue experiments using the AKT activator **SC79** and the PI3K inhibitor **LY294002**. **Results**: High *FTO* and *TNC* expression levels in clinical tissue samples correlate with poor patient survival. **Conclusions**: These findings reveal that the *FTO*/*TNC*/EMT axis represents a previously unrecognized epigenetic mechanism driving GC metastasis and suggest that targeting *FTO*-mediated m6A modification may provide a novel therapeutic strategy for GC patients with high metastatic potential.

## 1. Introduction

Gastric cancer (GC) is the fifth most prevalent cancer worldwide and a leading cause of cancer-related death, with approximately 60% of cases diagnosed at advanced stages due to the lack of early clinical symptoms [1,2]. Despite advancements in treatment options, the 5-year survival rate for GC remains low [3,4]. Cancer metastasis is a major contributor to poor prognosis and is a key factor in the failure of initial surgeries or the recurrence of lesions, necessitating further surgical interventions. The complex molecular mechanisms driving GC metastasis require deeper exploration. Therefore, the complex molecular mechanisms driving GC metastasis require deeper exploration.

Gastric cancer is a highly heterogeneous disease, encompassing distinct molecular subtypes with different histological differentiation patterns and clinical behaviors [5]. Intestinal-type GC often arises from chronic inflammation and progresses through a well-defined multistep cascade, whereas diffuse-type GC is characterized by poorly cohesive cells and a more aggressive clinical course. This heterogeneity is driven by diverse genetic and epigenetic alterations, including mutations in *TP53* and *CDH1*, as well as dysregulation of signaling pathways such as Wnt/β-catenin, TGF-β, and PI3K-AKT [6]. Understanding these complex mechanisms is essential for developing targeted therapeutic strategies.

The extracellular matrix (ECM) protein tenascin-C (TNC) has been implicated in promoting metastasis across various cancers [7,8,9,10]. In GC, *TNC* expression is elevated and correlates with poor prognosis [11]. However, the precise role of TNC in GC metastasis has not been fully explored. TNC is known to trigger epithelial-to-mesenchymal transition (EMT), a critical process in cancer metastasis in several types of cancer [9,12]. Therefore, understanding whether TNC contributes to GC metastasis by influencing EMT is crucial.

Both genetic and epigenetic mechanisms regulate *TNC* gene expression. The TNC promoter contains a TATA box and several transcription factors, including *Prrx1*, *Evx1*, and *SOX4*, which control its expression [10,13,14,15,16]. Epigenetically, *TNC* expression is modulated by c-Jun N-terminal kinase (JNK) signaling and suppressed by metastasis-suppressing microRNA *miR-335* [17,18]. However, the involvement of other epigenetic modifications in *TNC* regulation remains underexplored. In recent years, N6-methyladenosine (m6A) RNA methylation has emerged as a significant epigenetic modification involved in regulating gene expression, including mRNA splicing, decay, and translation [19,20]. m6A modification is regulated by methyltransferases (writers, e.g., methyltransferase-like 3 (METTL3) and methyltransferase-like 14 (METTL14)), demethylases (erasers, e.g., FTO, AlkB homolog 5 (ALKBH5)), and binding proteins (readers, e.g., YTH domain proteins) and plays a crucial role in tumor progression, metastasis, and chemotherapy resistance [20]. Despite its known regulatory impact on various malignancies, whether *TNC* mRNA is subjected to m6A modification remains unknown.

Here, we investigate TNC’s role in GC progression and hypothesize that an m6A demethylase is responsible for its upregulation. Through analysis of 33 m6A regulators, we identify FTO as the key regulator of TNC. Furthermore, we uncover YTHDF2 as the m6A reader that mediates *TNC* mRNA decay, and demonstrate that the PI3K-AKT pathway is essential for TNC/FTO-induced EMT. To our knowledge, this is the first study to link *TNC* expression to *FTO*-mediated m6A modification and reader-dependent mRNA stabilization, providing new insight into GC metastasis.

## 2. Materials and Methods

### 2.1. Cell Culture and Cell Line Development

Human gastric cancer cell lines, AGS (RRID: CVCL_0139), MGC-803 (RRID: CVCL_5334), and BGC-823 (RRID: CVCL_3360), were obtained from the American Type Culture Collection (ATCC). All human cell lines have been authenticated using STR (or SNP) profiling within the last three years. All cell lines tested negative for mycoplasma contamination using the MycoAlert Mycoplasma Detection Kit(Lonza, Basel, Switzerland). Cells were cultured in RPMI 1640 medium supplemented with 10% fetal bovine serum (FBS) and maintained at 37 °C in a humidified atmosphere containing 5% CO_2_.

AGS, MGC-803, and BGC-823 cell lines were selected to represent the molecular and pathological heterogeneity of gastric cancer. AGS is a well-differentiated intestinal-type gastric adenocarcinoma cell line with wild-type *p53*, whereas MGC-803 and BGC-823 are poorly differentiated cell lines derived from diffuse-type gastric cancer and harbor mutant *p53*, thus collectively covering diverse differentiation statuses and genetic backgrounds of GC.

### 2.2. Antibodies and Chemicals

Cell culture media and supplements, including fetal bovine serum (FBS), were purchased from Gibco(Waltham, MA, USA). All antibodies and chemical reagents used in this study are listed in Supplementary Table S1.

### 2.3. Western Blot Analysis

Cells were washed thrice with ice-cold phosphate-buffered saline (PBS) and lysed on ice for 30 min with NP40 buffer containing protease inhibitors. Protein concentrations were determined using a Bicinchoninic Acid (BCA) Protein Assay Kit (Beyotime Biotechnology, Shanghai, China). Equal amounts of protein (20 μg per lane) were separated by 10% sodium dodecyl sulfate-polyacrylamide gel electrophoresis (SDS-PAGE) and transferred to polyvinylidene difluoride (PVDF) membranes. The membranes were incubated overnight at 4 °C with the appropriate primary antibodies. Afterward, they were incubated with HRP-conjugated secondary antibodies at room temperature for two hours. Membrane-bound proteins were detected using a chemiluminescence detection kit. ImageJ software (version 1.54f, National Institutes of Health, Bethesda, MD, USA) was used for band density quantification. Three duplicates were performed for each group, and independent experiments were repeated three times.

### 2.4. Stable Cell Line Transfection and Establishment

To generate stable cell lines, recombinant lentiviruses (sh-scramble, sh-*TNC*, sh-*FTO*, or-*TNC*) were acquired from Genechem (Shanghai, China). Cells were transduced at a multiplicity of infection (MOI) of 10 in the presence of 8 μg/mL polybrene. After 24 h, the medium was replaced with fresh medium containing 2 μg/mL puromycin for selection, and stable transductants were maintained for 14 days. Control cells lacking the lentiviral vectors were maintained in parallel. Stable cell lines were confirmed by assessing protein and mRNA expression levels.

### 2.5. Transwell, Colony Formation, and Apoptosis Assays

In transwell migration assays, 50,000 cells were resuspended in a serum-free medium and seeded in the upper chambers of transwell inserts (8 μm pore size, Corning #3422) without Matrigel coating. The lower chambers contained 500 μL of complete medium. After incubation for 8–12 h, migrated cells were fixed in 4% paraformaldehyde for 15 min and stained with 0.1% crystal violet. For invasion assays, the upper chambers were pre-coated with Matrigel (50 μg/well; Corning #354234) before seeding the same number of cells. The number of migrated or invaded cells was counted under a microscope (five random fields per well).

Five hundred cells were seeded per well in 6-well plates for colony formation and incubated for two weeks. After incubation, colonies were fixed with 4% formaldehyde, stained with 0.1% crystal violet, and photographed.

Apoptosis was analyzed by treating cells with 5-fluorouracil (5-FU) for 24 h. Cells were harvested using 0.25% trypsin and stained with Annexin V-FITC/propidium iodide (PI) according to the manufacturer’s instructions. Apoptotic cells were analyzed using flow cytometry (BD Biosciences, San Jose, CA, USA; FACSAria).

The cellular experiments were run in triplicate and repeated three times for each group.

### 2.6. Scratch Migration Assay

A scratch migration assay was performed to assess cell migration. Cells were seeded in 6-well plates at 5 × 10^5^ cells per well. Once the cells reached 90% confluence, cells were treated with mitomycin C (10 μg/mL) for 2 h to inhibit cell proliferation. A scratch was made in the center of the cell monolayer using a P200 pipette tip(JET BIOFIL, Guangzhou, China). After removing floating cells by changing the medium, images were captured immediately and 24 h after incubation using a microscope at 10× magnification. Migration was quantified as percentage of wound closure = (width at 0 h − width at 24 h)/width at 0 h × 100%. The width of the scratch was measured to quantify cell migration. The cellular experiments were run in triplicate and repeated three times for each group.

### 2.7. Tissue Microarray and Immunohistochemistry (IHC)

All human subject studies were obtained with patients’ informed consent and performed under the guidelines and protocols approved by the ethical committee of Tongji Hospital, Tongji Medical College, HUST (IRB ID: 20230215). Expression of TNC and FTO was evaluated in human gastric cancer tissue microarrays containing 94 primary tumors and 86 adjacent normal tissues (HStmA180Su11, Shanghai Outdo Biotech Co., Ltd., Shanghai, China) from patients who did not receive preoperative chemotherapy or radiotherapy. After dewaxing and rehydration, antigen retrieval was performed using heat-induced epitope retrieval. The tissue sections were blocked with 10% donkey serum and incubated overnight at 4 °C with primary antibodies against TNC or FTO (diluted 1:100). Negative controls were performed by omitting the primary antibody. The secondary antibody was applied at room temperature for 2 h. Two independent pathologists evaluated staining, and the intensity and percentage of positively stained cells were scored to determine protein expression.

For immunocytochemistry, cells were seeded on glass coverslips and fixed with 4% paraformaldehyde for 15 min at room temperature. After permeabilization with 0.1% Triton X-100, cells were blocked with 1% bovine serum albumin (BSA) for 1 h. Primary antibodies were applied overnight at 4 °C, followed by incubation with secondary antibodies for 1 h at room temperature. Nuclei were stained with DAPI, and images were captured using a fluorescent microscope.

### 2.8. Animal Studies

All animal procedures were approved by the Institutional Animal Care and Use Committee (IACUC) of Tongji Hospital (IACUC ID: 202306026) and were carried out in accordance with institutional and national guidelines. Four-week-old female nude mice were purchased from Gem Pharmatech (Nanjing, China) and randomly assigned to experimental groups, with 5 mice per group. Mice were randomly allocated to groups, and all analyses (tumor measurement, nodule counting, IHC scoring) were performed blinded to group allocation. For subcutaneous xenograft tumor formation, 1 × 10^6^ cells were injected into the flank of each mouse. Tumor dimensions were measured every 3 days with a caliper, and tumor volume was calculated as (length × width^2^)/2. Tumors were harvested three weeks post-injection for further analysis. For tail vein metastasis assays, 5 × 10^6^ cells were injected into the tail veins of mice, and lungs were collected after two months for subsequent analysis. Metastatic nodules on the lung surface were counted blindly by two independent investigators.

### 2.9. m6A Methylated RNA Immunoprecipitation PCR (MeRIP-PCR)

Total RNA was extracted from sh*FTO* AGS and control cells using TRIzol (ThermoFisher, Waltham, MA, USA). One hundred micrograms of fragmented RNA (200 nt) was immunoprecipitated using 5 μg of anti-m6A antibody (riboMeRIP™ m6A Transcriptome Profiling Kit, RiboBio, Guangzhou, China) or control mouse IgG (negative control) at 4 °C for 2 h, followed by incubation with protein A/G magnetic beads for 2 h. After three washes with immunoprecipitation buffer, bound RNA was eluted and purified. RNA fragments were analyzed using quantitative real-time PCR (qRT-PCR) with primers listed in Table S1.

### 2.10. RNA Pull-Down Assay

To identify m6A reader proteins binding to *TNC* 3’UTR, we performed RNA pull-down using biotin-labeled RNA probes. Two probes covering the m6A site-3 of *TNC* 3’UTR were designed: (1) m6A probe—containing m6A modification at the consensus site; (2) a probe—same sequence with unmodified adenosine (negative control). Probes were biotinylated at the 3’ end. Cells (AGS, shNC or sh*FTO*) were lysed in NP40 lysis buffer (50 mM Tris-HCl pH 7.4, 150 mM NaCl, 1% NP-40, protease and RNase inhibitors). An amount of 1 mg of total protein lysate was incubated with 50 pmol of each probe at 4 °C for 2 h, followed by incubation with streptavidin magnetic beads (ThermoFisher) for 1 h. Beads were washed three times with lysis buffer, and bound proteins were eluted by boiling in SDS loading buffer. Western blotting was performed to detect YTHDF2, IGF2BP1, IGF2BP2, and IGF2BP3.

### 2.11. mRNA Stability Assay

AGS cells with indicated genetic backgrounds (shNC or sh*FTO*1), si*YTHDF2*, sh*FTO* + si*YTHDF2*, sh*FTO* + *TNC* OE) were plated in 6-well plates. At 70–80% confluence, cells were treated with actinomycin D (5 μg/mL). Total RNA was harvested at 0, 1, 2, and 3 h post-treatment. *TNC* mRNA levels were quantified by qRT-PCR (normalized to *ACTB*) and expressed as percentage of remaining mRNA relative to time 0. Half-life (t1/2) was calculated using the decay curve fit to a one-phase exponential decay model (GraphPad Prism).

### 2.12. PI3K-AKT Pathway Rescue Experiments

AGS cells (shNC or sh*FTO*1) were treated with DMSO (vehicle), **SC79** (AKT activator, 4 μg/mL), or **SC79** + **LY294002** (PI3K inhibitor, 10 μM) for 24 h. **SC79** was added 1 h before **LY294002** in the combination group. Cells were then harvested for Western blot, Transwell migration, and wound healing assays as described above. All treatments were performed in triplicate.

### 2.13. Real-Time Quantitative Polymerase Chain Reaction (qPCR)

Total RNA was extracted using the RNA isolator reagent, and cDNA was synthesized using the HiScript II 1st Strand cDNA Synthesis Kit (Vazyme Biotech, Nanjing, China). Real-time PCR was performed using ChamQ SYBR qPCR Master Mix on an ABI 7300 Real-Time PCR System (Applied Biosystems, Foster City, CA, USA). *ACTB* was used as the single reference gene after validating its stable expression across all experimental conditions (tested by preliminary experiments). The experiments were run in triplicate and repeated three times. Gene-specific primers, designed from the PrimerBank database (https://pga.mgh.harvard.edu/primerbank/), are provided in Table S1.

### 2.14. Luciferase Reporter and Mutagenesis Assays

The psiCHECK-2 dual-luciferase vector was purchased from Promega. Genomic DNA from TNC was amplified by PCR and inserted into the psiCHECK-2 vector using XhoI and NotI enzymes. Potential m6A binding sites within the 3’ untranslated region (UTR) of TNC were identified, and mutations were introduced using the Q5 Site-Directed Mutagenesis Kit (New England Biolabs, Ipswich, MA, USA, #E0554). Luciferase activity was measured using the Dual-Luciferase Reporter Gene Assay Kit (Promega, Madison, WI, USA, catalog number E1901).

### 2.15. Public Database and Bioinformatics Analysis

RNA sequencing (RNA-seq) expression profiles and corresponding clinicopathological and survival information for stomach adenocarcinoma were obtained from the TCGA-STAD cohort. TCGA gene expression values were normalized as log2(TPM + 1). GEO expression matrices and corresponding platform annotation files were downloaded from the Gene Expression Omnibus database. For GEO microarray datasets, probes were mapped to official gene symbols according to the corresponding platform annotation files; when multiple probes mapped to the same gene, the mean expression value was used for downstream analysis. Samples without available survival information were excluded from the corresponding survival analyses.

For survival analyses, patients in each cohort were divided into high- and low-TNC expression groups using the cohort-specific median *TNC* expression level as the cutoff. Patients with *TNC* expression above the median were classified as the high-*TNC* expression group, whereas patients with TNC expression at or below the median were classified as the low-*TNC* expression group. Overall survival (OS), disease-free survival (DFS), and progression-free survival (PFS) were analyzed when available. Kaplan–Meier survival curves were generated, and survival differences between groups were evaluated using two-sided log-rank tests. Hazard ratios (HRs) and 95% confidence intervals (CIs) were estimated using univariate Cox proportional hazards regression, with the *TNC*-low group used as the reference group.

To explore biological pathways associated with TNC expression, TCGA-STAD tumor samples were divided into *TNC*-high and *TNC*-low groups according to the median *TNC* expression level. Differential expression analysis between the two groups was performed at the transcriptome-wide level. For gene set enrichment analysis (GSEA), genes retained after preprocessing were ranked according to their signed differential expression statistic between the *TNC*-high and *TNC*-low groups. The ranked gene list was then used as the input for GSEA. Curated pathway gene sets were obtained from the KEGG *Homo sapiens* pathway database and the MSigDB Hallmark gene sets. The enrichment score (ES), normalized enrichment score (NES), nominal *p* value, and false discovery rate (FDR) q value were used to evaluate pathway enrichment.

For Kyoto Encyclopedia of Genes and Genomes (KEGG) pathway enrichment analysis, *TNC*-associated differentially expressed genes were used as the input gene list. Gene symbols were converted to Entrez Gene IDs before enrichment analysis. Differential expression analysis was performed using the limma R package (version 3.50.0). Genes with |log2FC| > 1 and adjusted *p* < 0.05 were considered significantly differentially expressed. KEGG pathway enrichment was performed using the clusterProfiler R package (version 4.2.0) against the KEGG Homo sapiens database (release 104.0). *p* values were calculated using a hypergeometric test and adjusted for multiple comparisons using the Benjamini–Hochberg false discovery rate method. Pathways with adjusted *p* values < 0.05 were considered statistically significant. Gene set enrichment analysis (GSEA, version 4.1.0) was performed with 1000 gene set permutations, using the Molecular Signatures Database (MSigDB) Hallmark gene set (version 7.5.1); FDR < 0.25 was considered significant. In the KEGG dot plot, GeneRatio was defined as the number of input genes annotated to a given KEGG pathway divided by the total number of input genes used for enrichment analysis. Dot size represents the number of enriched genes, and dot color represents the −log10-transformed adjusted *p* value.

### 2.16. Statistical Analysis

Statistical analysis was performed using GraphPad Prism 10.1.2, R software (v4.0.0), and SPSS 25.0 (IBM Corporation, Armonk, NY, USA). Continuous variables are presented as mean ± standard deviation (SD). Comparisons between two groups were performed using two-tailed Student’s *t*-test. Comparisons among multiple groups were performed using one-way one-way analysis of variance (ANOVA) followed by Tukey’s post-hoc test for multiple comparisons. Normality was verified using Shapiro–Wilk test, and homogeneity of variance by Levene’s test. Fisher’s exact test was used for categorical data. For survival analyses, overall survival (OS), disease-free survival (DFS), and progression-free survival (PFS) were analyzed when available. Survival curves were generated using the Kaplan–Meier method, and survival differences between groups were evaluated using two-sided log-rank tests (univariate analyses, Table 1). Hazard ratios (HRs) and 95% confidence intervals (CIs) were estimated using multivariate Cox proportional hazards regression (Table 1). For pathway enrichment analyses (KEGG and GSEA), *p* values were adjusted for multiple testing using the Benjamini–Hochberg false discovery rate method, and adjusted *p* values < 0.05 were considered statistically significant. For all other analyses, a *p* value < 0.05 was considered statistically significant.

## 3. Results

### 3.1. TNC Overexpression in GC Tissues and Its Association with Poor Prognosis

**Figure 1:**
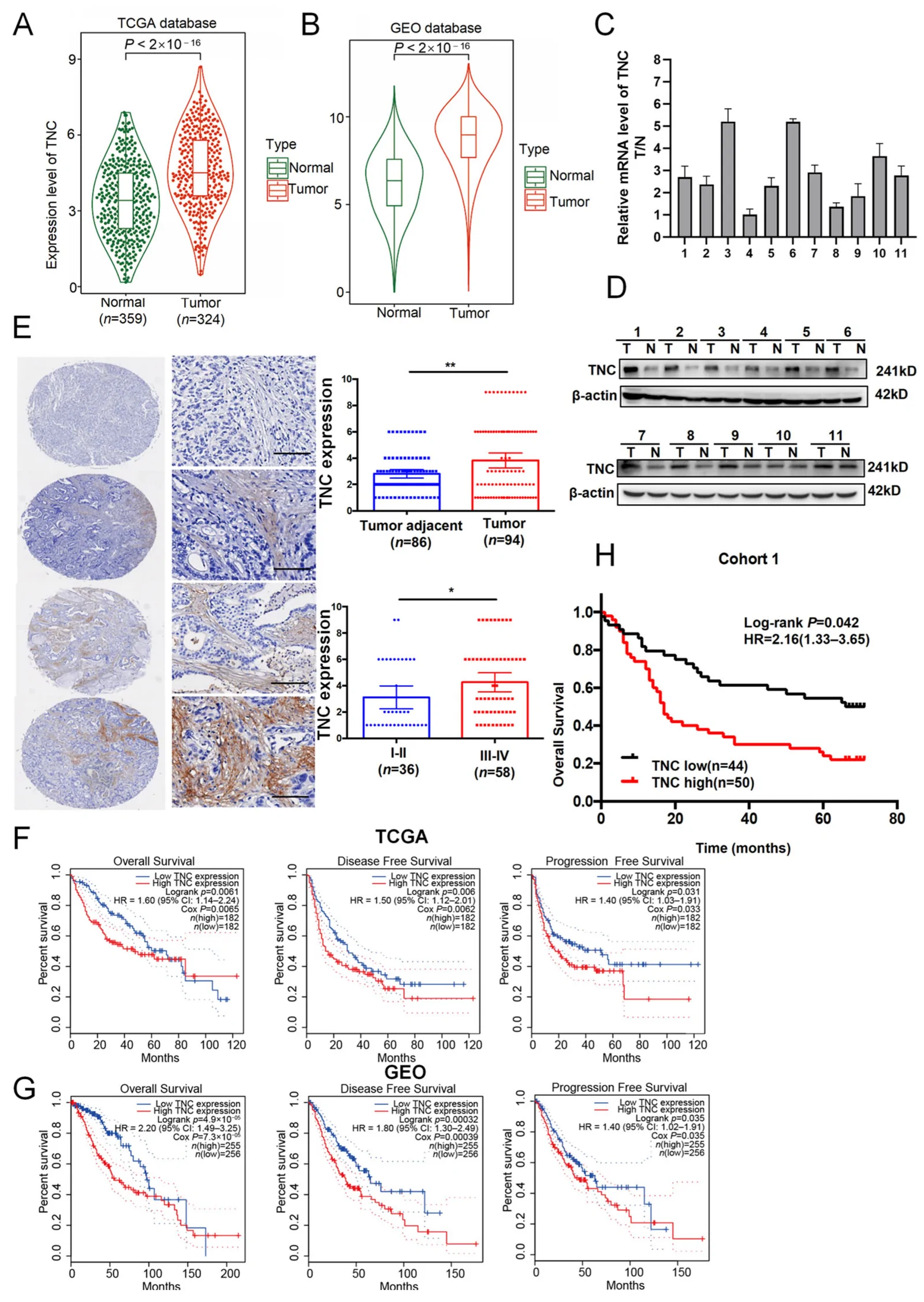
TNC overexpression and its association with tumor survival in GC patients. (**A**) *TNC* expression levels in the TCGA-STAD (stomach adenocarcinoma) database for gastric cancer. (**B**) *TNC* expression levels in GEO datasets for gastric cancer. (**C**) *TNC* mRNA expression in tumor versus adjacent normal tissue samples; T, tumor; N, adjacent normal. (**D**) TNC protein expression levels in tumor and adjacent normal tissues. T, tumor; N, adjacent normal. (**E**) TNC immunohistochemical staining in paired clinical specimens with statistical quantification. 94 cases from tumor tissue and 86 cases from adjacent normal tissue. TNC levels in patients at different clinical stages: 36 cases in stage I–II and 58 cases in stage III–IV. Scale bar: 50 μm (**F**) Kaplan–Meier survival analysis of overall survival, disease-free survival, and progression-free survival based on *TNC* mRNA expression levels in TCGA-STAD datasets. Patients were stratified into high- and low-*TNC* expression groups using the cohort-specific median *TNC* expression level as the cutoff. Patients with *TNC* expression above the median were assigned to the high-*TNC* expression group, and those with *TNC* expression at or below the median were assigned to the low-*TNC* expression group. Solid lines represent the Kaplan–Meier estimates of survival, and dashed lines indicate the corresponding 95% confidence intervals. The log-rank test was employed to determine *p*-values. (**G**) Kaplan–Meier survival analysis for overall survival, disease-free survival, and progression-free survival based on *TNC* mRNA expression levels in GEO datasets. Patients were stratified using the same median-based cutoff strategy, and *p*-values were determined using the log-rank test. (**H**) Kaplan–Meier survival analysis of overall survival (OS) based on TNC expression levels in 94 GC patients from tissue microarrays (Cohort 1). Patients were stratified into high- and low-TNC expression groups using the median IHC score as the cutoff. The log-rank test was employed to determine *p*-values. * *p* < 0.05; ** *p* < 0.01

We observed significant overexpression of *TNC* in gastric cancer (GC) tissues. Analysis of clinical datasets from TCGA (The Cancer Genome Atlas) and GEO databases revealed a marked upregulation of *TNC* mRNA in GC tissues compared to normal tissues (Figure 1A,B). Further validation in a cohort of 11 paired GC tissues (Cohort 2) via qRT-PCR and Western blot confirmed elevated *TNC* mRNA and protein levels in GC tissues relative to adjacent normal tissues (Figure 1C,D). Immunohistochemical (IHC) analysis of tissue microarrays (TMAs) from 94 GC patients (Cohort 1) demonstrated predominant localization of TNC in the extracellular matrix, with significantly higher expression in gastric tumor tissues (Figure 1E). Furthermore, advanced-stage (III–IV) GC tissues exhibited increased TNC expression compared to early-stage (I–II) tumors, and TNC levels correlated with the American Joint Committee on Cancer (AJCC) stage (Table S2). Survival analysis of TCGA and GEO datasets indicated that patients with high *TNC* expression had significantly poorer survival rates (Figure 1F,G), which was corroborated by TMA analysis from our patient cohort (Figure 1H). Univariate survival analysis using the log-rank test revealed that AJCC stage (*p* < 0.001), venous invasion (*p* = 0.001), nervous invasion (*p* = 0.016), and *TNC* expression (*p* = 0.003) were significantly associated with overall survival (OS) in GC patients (Table 1). These variables were then entered into a multivariate Cox regression model. Multivariate analysis demonstrated that AJCC stage (HR = 2.722, 95% CI = 1.449–5.110, *p* = 0.002) and *TNC* expression (HR = 1.734, 95% CI = 1.008–2.982, *p* = 0.047) remained independent prognostic factors for OS, whereas venous invasion and nervous invasion lost statistical significance (Table 1). These findings suggest that *TNC* overexpression is a marker of poor prognosis in GC.

### 3.2. TNC Knockdown Inhibits GC Cell Proliferation, Migration, Invasion, and Tumor Progression In Vivo

**Figure 2:**
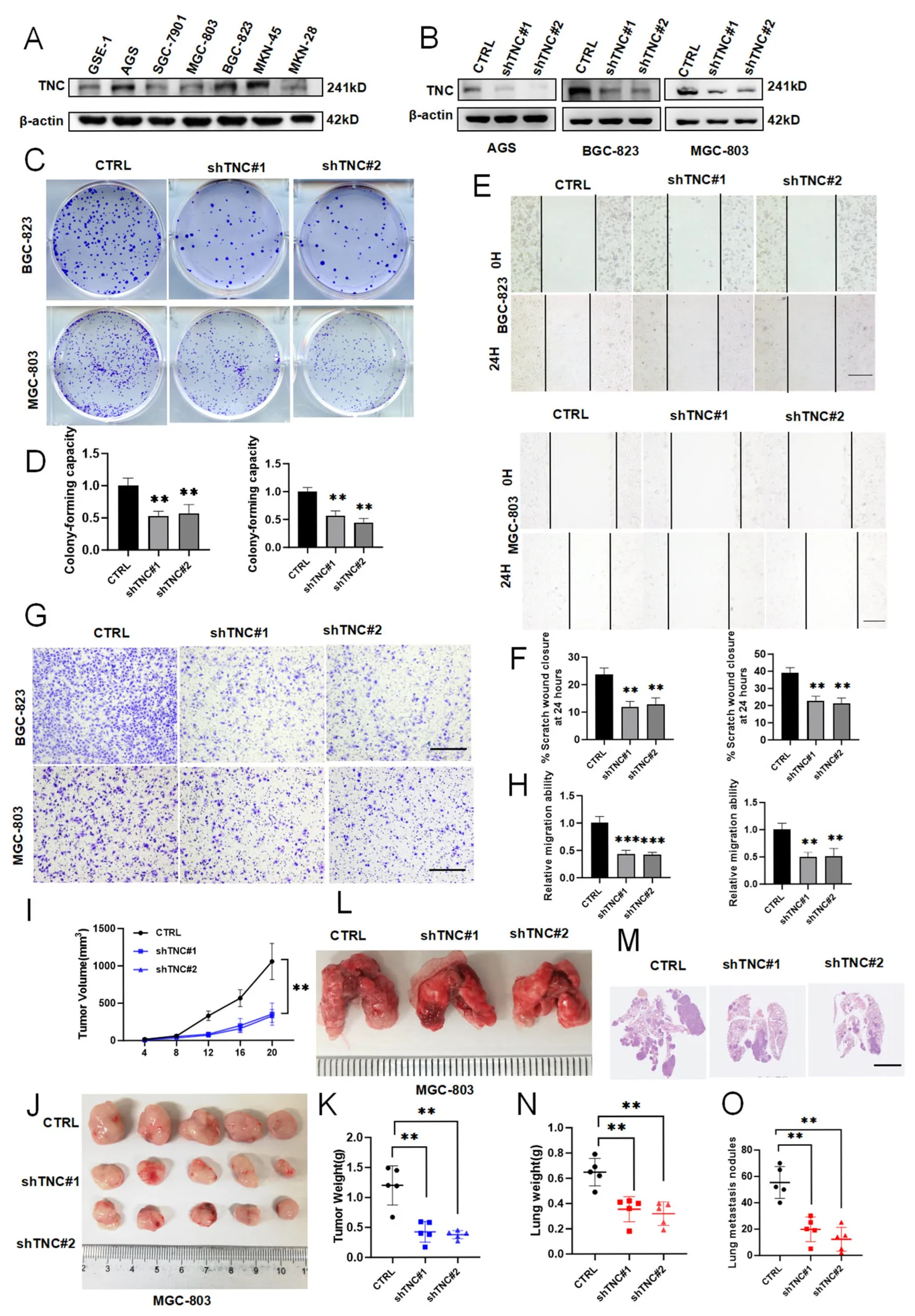
TNC knockdown inhibits proliferation, apoptosis, invasion, and migration of GC cells. (**A**) Western blot analysis of TNC expression in seven GC cell lines (GES-1, AGS, SGC-7901, MGC-803, BGC-823, MKN-45, MKN-28), with *ACTB* as the internal control. (**B**) Verification of *TNC* knockdown in AGS, BGC-823, and MGC-803 cells via Western blot. (**C**,**D**) Colony formation assays in MGC-803 and BGC-823 cells with control (CTRL) or sh*TNC*. Representative images from three biological replicates (**C**) and quantified (**D**). (**E**,**F**) Wound healing assays in BGC-823 and MGC-803 cells with control (CTRL) or sh*TNC*. Representative images from three biological replicates (**E**) and quantified (**F**). Scale bar: 100 μm. (**G**,**H**) Transwell invasion assays in BGC-823 and MGC-803 cells with control (CTRL) or sh*TNC*. Representative images from three biological replicates (**G**) and quantified (**H**). Scale bar: 200 μm. (**I**–**K**) Tumor growth analysis in nude mice injected subcutaneously with MGC-803 *TNC* knockdown cells (sh*TNC*) or control cells (CTRL). Tumor volume was measured three weeks after implantation, and growth curves were plotted based on tumor volumes (**I**). Representative images of tumors (**J**), and tumor weights (**K**). (**L**–**O**) Lung metastasis analysis in nude mice injected with MGC-803 *TNC* knockdown cells (sh*TNC*) or control cells (CTRL) via tail vein. Representative lung metastasis images (**L**) and H&E staining of lung tissue (**M**). Scale bar: 5 mm. Quantitative analysis of metastatic lung nodules (**O**) and lung weights (**N**). Data shown as mean ± SD. Statistical analyses were performed using Student’s *t*-test. ** *p* < 0.01; *** *p* < 0.001.

Western blot analysis of seven GC cell lines and the human gastric epithelial cell line GES-1 identified AGS, BGC-823, and MGC-803 as high TNC expressers (Figure 2A). These cell lines were transfected with two different shRNA lentiviruses targeting *TNC* (sh*TNC*#1 and sh*TNC*#2), with successful knockdown confirmed at both mRNA and protein levels (Figure 2B and Supplementary Figure S1A). Meanwhile, we also established *TNC* high-expression cell lines using two different *TNC* overexpression lentiviruses (*TNC* OE#1 and *TNC* OE#2) and confirmed by mRNA and protein level analysis (Supplementary Figure S2A,B). *TNC* knockdown significantly reduced colony formation efficiency in AGS, BGC-823, and MGC-803 cells compared to control cells (Figure 2C,D and Supplementary Figure S1D). The number of cell clones formed was increased in *TNC* overexpressing cells (Supplementary Figure S2C). Flow cytometry analysis revealed no significant increase in Annexin V-positive cells upon *TNC* knockdown, suggesting that *TNC* silencing inhibits proliferation but not cell survival in GC cells (Supplementary Figure S1B,C). Wound healing assays showed that *TNC* knockdown significantly impaired the migratory capacity of AGS, BGC-823, and MGC-803 cells (Figure 2E,F and Supplementary Figure S1E). In *TNC* overexpressing cells, wound healing assays showed that *TNC* overexpression significantly promoted the migratory capacity (Supplementary Figure S2D). Transwell invasion assays confirmed that *TNC* silencing dramatically suppressed the invasive ability of GC cells (Figure 2G,H and Supplementary Figure S1F). In *TNC* overexpressing cells, Transwell invasion assays confirmed that *TNC* overexpression significantly enhanced the invasive ability (Supplementary Figure S2E).

In vivo studies using subcutaneous injections of stable *TNC*-knockdown MGC-803 cells into 4-week-old female Balb/c-nude mice. Growth curve analysis indicated that tumors in the sh*TNC* group exhibited slower progression (Figure 2I). After 20 days, mice were sacrificed, and the xenografts were collected and weighed. Tumors derived from sh*TNC* cells were smaller and significantly lighter in weight compared to controls (Figure 2J,K). Additionally, to determine whether *TNC* knockdown abrogate tumor metastasis in vivo, we injected CTRL and sh*TNC* cells into the tail veins of nude mice. The results showed that sh*TNC* cells generated fewer metastatic nodules and lower tumor weights than did CTRL cells (Figure 2L–O). These results demonstrate that *TNC* silencing inhibits tumor growth and metastasis both in vitro and in vivo.

### 3.3. TNC Knockdown Inhibits EMT via PI3K-Akt Signaling

**Figure 3:**
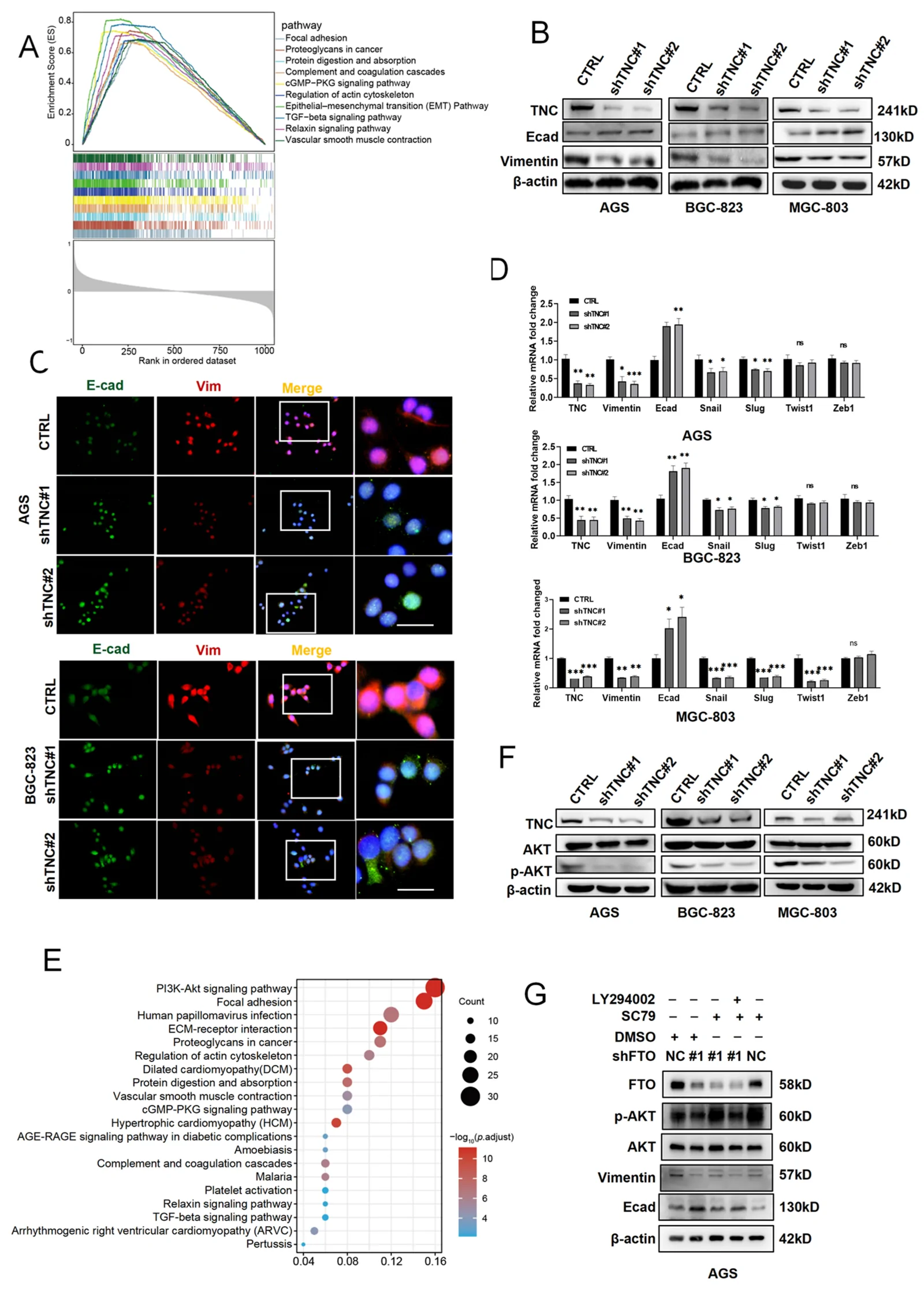
TNC knockdown inhibits EMT through the PI3K-akt signaling pathway in GC cells. (**A**) GSEA of TCGA-STAD RNA-seq data using a *TNC*-associated ranked gene list. The y-axis shows the enrichment score, which represents the running-sum statistic indicating whether genes from a given pathway are enriched at the top or bottom of the ranked gene list. Vertical tick marks indicate the positions of pathway genes in the ranked list, and the lower panel shows the ranked metric across the ordered dataset. Colors indicate individual pathways as shown in the legend. (**B**) Protein levels of TNC, E-cadherin (Ecad), and vimentin in AGS, BGC-823, and MGC-803 cells with control (CTRL) or TNC knockdown (shTNC) analyzed by Western blot. *ACTB* as the internal control. (**C**) Immunofluorescence staining of E-cadherin (green) and vimentin (red) in AGS, BGC-823, control (CTRL), and sh*TNC* cells. Nuclei stained with DAPI (blue). Scale bar: 50 μm. (**D**) mRNA expression levels of *TNC*, vimentin, E-cadherin, and EMT markers (*Snail, Slug, Twist1, Zeb1*) in AGS, BGC-823, and MGC-803 cells with control (CTRL) or TNC knockdown (sh*TNC*) by qPCR. *ACTB* as the internal control. (**E**) KEGG pathway enrichment analysis of *TNC*-associated genes. The x-axis represents GeneRatio, defined as the number of input genes annotated to a given KEGG pathway divided by the total number of input genes used for enrichment analysis. Dot size indicates the number of enriched genes, and dot color indicates the −log10-transformed adjusted *p* value. *p* values were adjusted for multiple comparisons using the Benjamini–Hochberg false discovery rate method. (**F**) Protein levels of TNC, AKT, and p-AKT in AGS, BGC-823, and MGC-803 cells with control (CTRL) or *TNC* knockdown (sh*TNC*) analyzed by Western blot. *ACTB* as the internal control. (**G**) Western blot analysis of AKT, p-AKT, vimentin, and E-cadherin in sh*FTO* AGS cells treated with DMSO (vehicle), **SC79** (AKT activator), or **SC79** + **LY294002** (PI3K inhibitor). *ACTB* served as the loading control. *p < 0.05; **p < 0.01; ***p < 0.001; ns, no significant.

TNC has been implicated in promoting EMT in breast cancer, but its role in GC EMT has not been well characterized. Gene set enrichment analysis (GSEA) of TCGA datasets revealed a strong association between *TNC* expression and EMT (Figure 3A). We used E-cadherin and vimentin as indicators to detect EMT in gastric cancer (GC) cell lines, and verified the results through Western blot, immunofluorescence (IF), and qRT-PCR experiments. The results showed an increase in E-cadherin expression and a decrease in vimentin expression (Figure 3B–D). In *TNC* overexpressing cells, the results showed a decreased in E-cadherin expression and an increased in vimentin expression (Supplementary Figure S2B). Phalloidin staining showed reduced actin stress fibers upon *TNC* knockdown (Supplementary Figure S3A). More importantly in vivo experiments showed that E-cadherin expression increased and vimentin expression decreased in tumors from *TNC* knockdown mice, as observed through Western blot and IHC (Supplementary Figure S3B,C). Additionally, mRNA levels of EMT transcription factors, including *Snail, Slug*, and *Twist1*, were downregulated following *TNC* silencing (Figure 3D). To explore the downstream signaling pathways activated by TNC-induced EMT, KEGG pathway enrichment analysis identified the PI3K-AKT pathway as a major target (Figure 3E). The results showed that phosphorylation of AKT was significantly reduced in *TNC* knockdown cells (Figure 3F), indicating that TNC induces EMT through the PI3K-AKT signaling pathway.

We next asked whether PI3K-AKT activation is required for TNC-induced EMT. Using *FTO* knockdown as a model that reduces *TNC* expression, we treated sh*FTO* AGS cells with the AKT activator **SC79**. **SC79** restored p-AKT levels, reduced E-cadherin, and increased vimentin expression (Figure 3G). Co-treatment with the PI3K inhibitor **LY294002** blocked these effects. Wound healing assays (Figure S4A,B) and transwell migration assays (Figure S4C,D) showed that **SC79** significantly rescued the migratory and invasive defects caused by *FTO* knockdown, and **LY294002** again reversed the rescue. In control shNC cells, **SC79** alone enhanced migration and EMT markers. These results demonstrate that PI3K-AKT signaling is necessary and sufficient for the EMT and migration induced by the *FTO*/*TNC* axis.

### 3.4. TNC Positively Correlates with FTO Expression in GC

**Figure 4:**
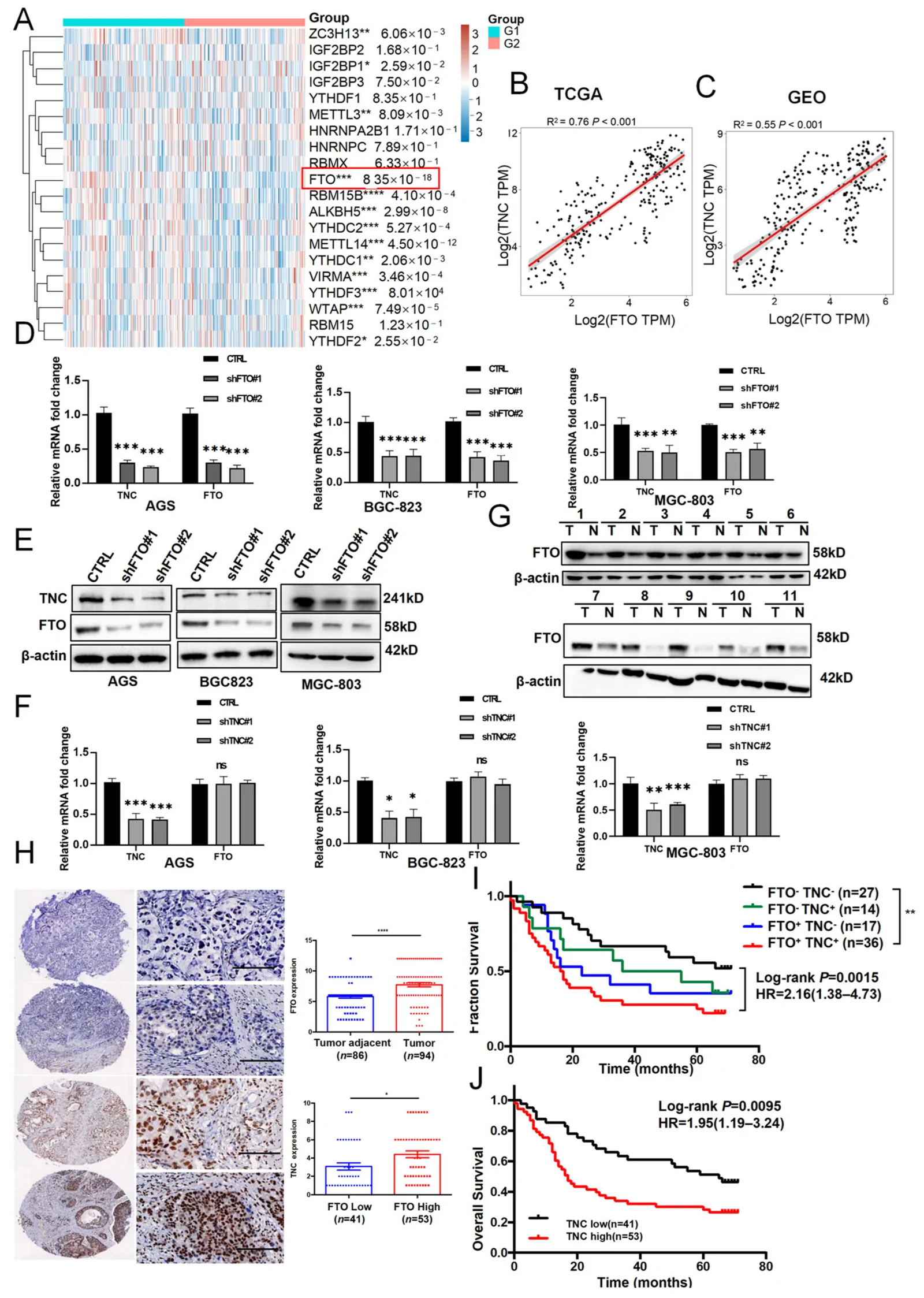
TNC positively correlates with FTO expression in gastric cancer. (**A**) Heatmap showing the Pearson correlation coefficients between each m6A-related gene and *TNC* expression across TCGA-STAD samples. (**B**) Correlation of *TNC* and *FTO* expression in TCGA-STAD dataset (*n* = 211). Log2(TPM) values are shown. (**C**) Correlation of *TNC* and *FTO* expression in GEO datasets (*n* = 211). Log2(TPM) values are shown. (**D**) mRNA expression levels of *TNC* and *FTO* in AGS, BGC-823, and MGC803 cells (CTRL and sh*FTO* cells). (**E**) TNC, FTO protein expression in AGS, BGC-823, and MGC-803 cells with stable *FTO* knockdown (sh*FTO*) analyzed by Western blot. *ACTB* as internal control. (**F**) mRNA levels of *TNC* and *FTO* in AGS, BGC-823, and MGC803 negative control (CTRL) and sh*TNC* cells. *ACTB* as internal control. (**G**) FTO protein expression levels in tumor and adjacent normal tissues. T, tumor; N, adjacent normal. (**H**) FTO immunohistochemical staining in paired clinical specimens with statistical quantification. IHC scores for FTO expression in normal and tumor tissues (upper right images). TNC expression in GC patients with high FTO (*n* = 53) and low FTO (*n* = 41) expression (lower right images). Scale bar: 50 μm. Data shown as mean±SEM. Statistical analyses were performed using unpaired *t*-test. (**I**) Kaplan–Meier survival analysis based on *FTO* expression in 94 GC patients (27 with *FTO*^−^ TNC^−^, 14 with *FTO*^−^*TNC*^+^, 14 with *FTO*^+^*TNC*^−^, and 36 with *FTO*^+^*TNC*^+^). Median-based cutoff for *FTO* expression used, and log-rank test was performed. (**J**) Kaplan–Meier curves of overall survival comparing GC patients with low and high FTO expression levels. High: IHC score > 7, *n* = 53; low: IHC score < 7, *n* = 41. *p* = 0.0095.* *p* < 0.05; ** *p* < 0.01; *** *p* < 0.001; **** *p* < 0.0001; ns, not significant.

To investigate whether *TNC* mRNA is regulated through m6A methylation, we analyzed the relationship between m6A-related genes and *TNC* expression using TCGA datasets. Among the 20 m6A-related genes, FTO exhibited the strongest positive correlation with *TNC* expression (Figure 4A), suggesting that FTO may regulate *TNC* through m6A modification. Further analysis of clinical datasets confirmed that FTO demonstrated the most significant positive correlation with *TNC* in both the TCGA (Pearson r = 0.76, Figure 4B) and GEO (Pearson r = 0.55, Figure 4C) databases. To investigate the regulatory mechanism of FTO on *TNC*, we transfected three GC cell lines with shRNA lentiviruses targeting *FTO* (sh*FTO*#1 and sh*FTO*#2), resulting in efficient knockdown of FTO at both mRNA and protein levels (Figure 4D,E). Consistent with TCGA data, the knockdown of *FTO* decreased *TNC* expression at both mRNA and protein levels (Figure 4D,E). Notably, *TNC* knockdown did not affect *FTO* mRNA levels (Figure 4F), suggesting that *FTO* acts upstream of *TNC*. Further studies revealed that similar to *TNC* knockdown, *FTO* knockdown significantly suppressed the proliferation, migration, and invasion of GC cells (Supplementary Figure S5A,D,E). The cell apoptosis experiments suggested that *FTO* silencing inhibits proliferation but not cell survival in GC cells (Supplementary Figure S5B,C). By measuring the RNA levels of EMT markers, the results showed that *FTO* knockdown downregulated the expression of vimentin, *Snail, Slug*, and *Twist1*, while E-cadherin expression was upregulated (Figure S6A). Western blot and IF experiments also observed that E-cadherin expression increased and vimentin expression decreased in *FTO* knockdown cells (Figure S6B,C). IHC staining confirmed elevated FTO expression in GC tissues, positively correlating with *TNC* expression (Figure 4H). Survival analysis indicated that high FTO expression was associated with significantly poorer overall survival (OS) in GC patients (Figure 4I) with the worst prognosis observed in patients with high *TNC* and *FTO* expression (Figure 4J). Western blot analysis of 11 randomly selected GC tissues from Cohort 2 also demonstrated upregulation of FTO, similar to *TNC* expression (Figure 4G). These results suggest that *FTO* regulates TNC expression and contributes to GC progression.

### 3.5. FTO Regulates TNC via m6A Demethylation and YTHDF2 Mediates TNC mRNA Decay

**Figure 5:**
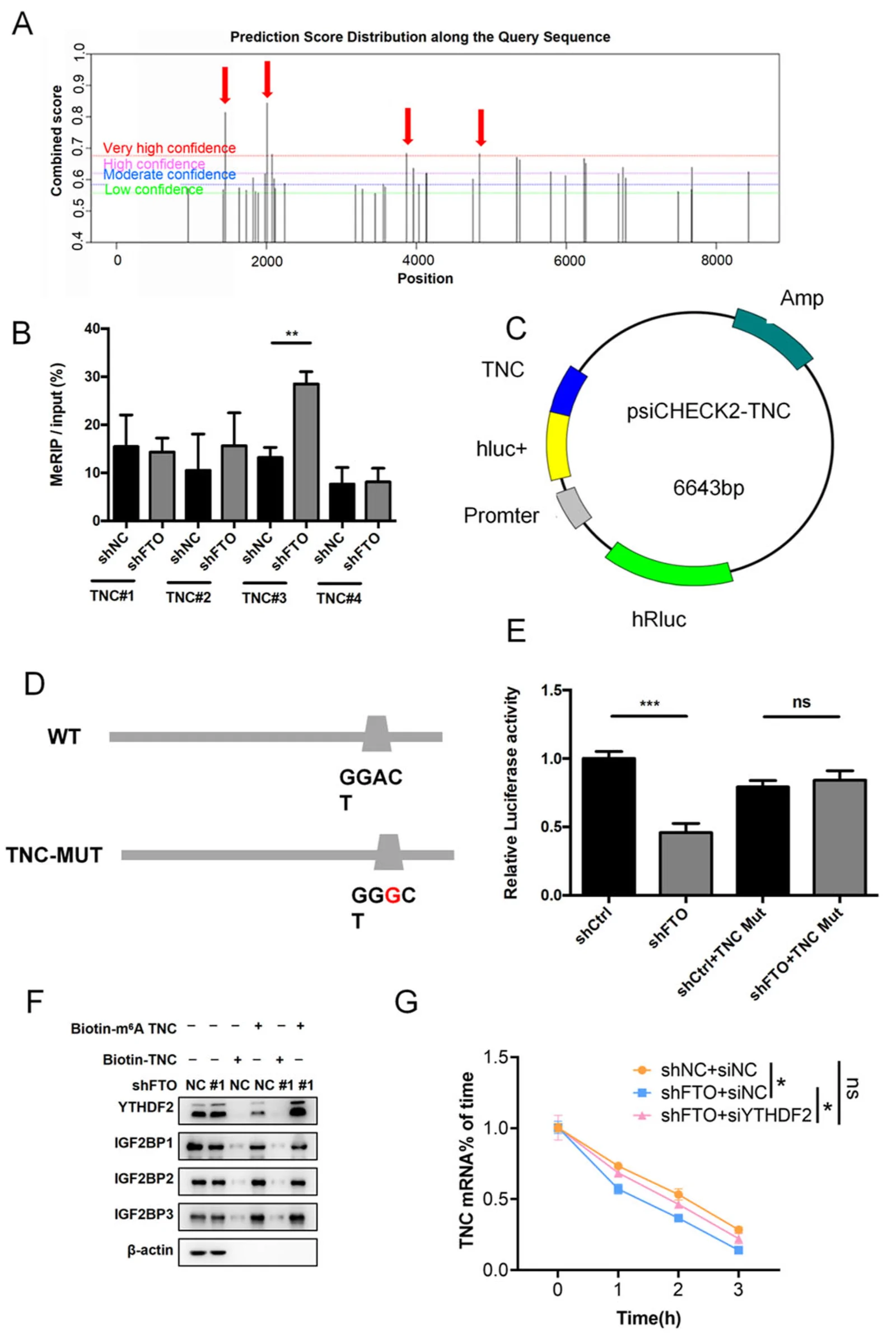
Epigenetic silencing of TNC via FTO-mediated m6A modification. (**A**) Prediction of four potential m6A consensus sequence motifs in the *TNC* mRNA 3’UTR using the m6A modification site predictor (https://www.cuilab.cn/m6asiteapp/old, accessed on 1 June 2024). (**B**) m6A MeRIP analysis showing that *FTO* knockdown increases m6A methylation in *TNC* mRNA. ** *p* ≤ 0.01. (**C**) A firefly luciferase reporter construct was fused with wild-type or m6A consensus sequence mutant *TNC* 3’UTR. (**D**) m6A consensus sequence mutations created by replacing cytosine with guanine. (**E**) Analysis of relative luciferase activity of wild-type and mutant *TNC* reporter vectors in *FTO*-knockdown AGS cells. (**F**) RNA pull-down assay showing the binding of m6A reader proteins to the biotinylated *TNC* 3’UTR probes (m6A-modified or unmodified A probe) in AGS cells with control (shNC) or FTO knockdown (sh*FTO*). Bound proteins were detected by Western blot using antibodies against YTHDF2 and IGF2BP1-3. (**G**) mRNA stability of *TNC* assessed by actinomycin D chase assay in AGS cells with the indicated genetic backgrounds (shNC, sh*FTO*, and sh*FTO* + si*YTHDF2*). Total RNA was harvested at the indicated time points after actinomycin D treatment, and *TNC* mRNA levels were quantified by qRT-PCR and normalized to *ACTB*. Data are presented as percentage of remaining mRNA relative to time 0 (mean±SD, *n* = 3 biological replicates). Statistical comparisons of half-lives were performed using one-way ANOVA with Tukey’s post-hoc test; * *p* < 0.05; ** *p* < 0.01; *** *p* < 0.001; ns, not significant.

To further confirm that *TNC* is a target of FTO-mediated m6A modification, we performed m6A-RNA immunoprecipitation assays followed by qRT-PCR. Four potential m6A consensus sites in the *TNC* 3’UTR were predicted (Figure 5A). *FTO* knockdown increased m6A modification at site-3 of *TNC* mRNA (Figure 5B). We further explored the effect of m6A modification on *TNC* expression by introducing mutations at the predicted m6A site (site-3) in the *TNC* 3’UTR. Luciferase assays demonstrated that *FTO* knockdown did not change *TNC* methylation site-3 mutant luciferase activity, confirming that FTO-mediated m6A modification regulates *TNC* expression at site-3 (Figure 5C–E).

We then identified the m6A reader responsible for *TNC* mRNA stability. RNA pull-down assays using biotinylated probes spanning *TNC* 3’UTR site-3 showed that YTHDF2, but not IGF2BP family proteins, selectively bound the m6A-modified probe in control AGS cells. The unmodified A probe showed no significant binding. In *FTO* knockdown cells, YTHDF2 binding to the m6A probe was markedly enhanced (Figure 5F), consistent with elevated m6A levels. Actinomycin D chase assays revealed that *FTO* knockdown significantly shortened the half-life of *TNC* mRNA compared to control cells; conversely, *YTHDF2* knockdown alone prolonged *TNC* mRNA stability. Notably, combined knockdown of *FTO* and *YTHDF2* significantly restored *TNC* mRNA stability (Figure 5G). These data confirm that FTO-mediated m6A demethylation reduces YTHDF2 binding, thereby enhancing *TNC* mRNA stability.

### 3.6. FTO-Mediated Epigenetic Activation of TNC Promotes EMT and GC Progression

**Figure 6:**
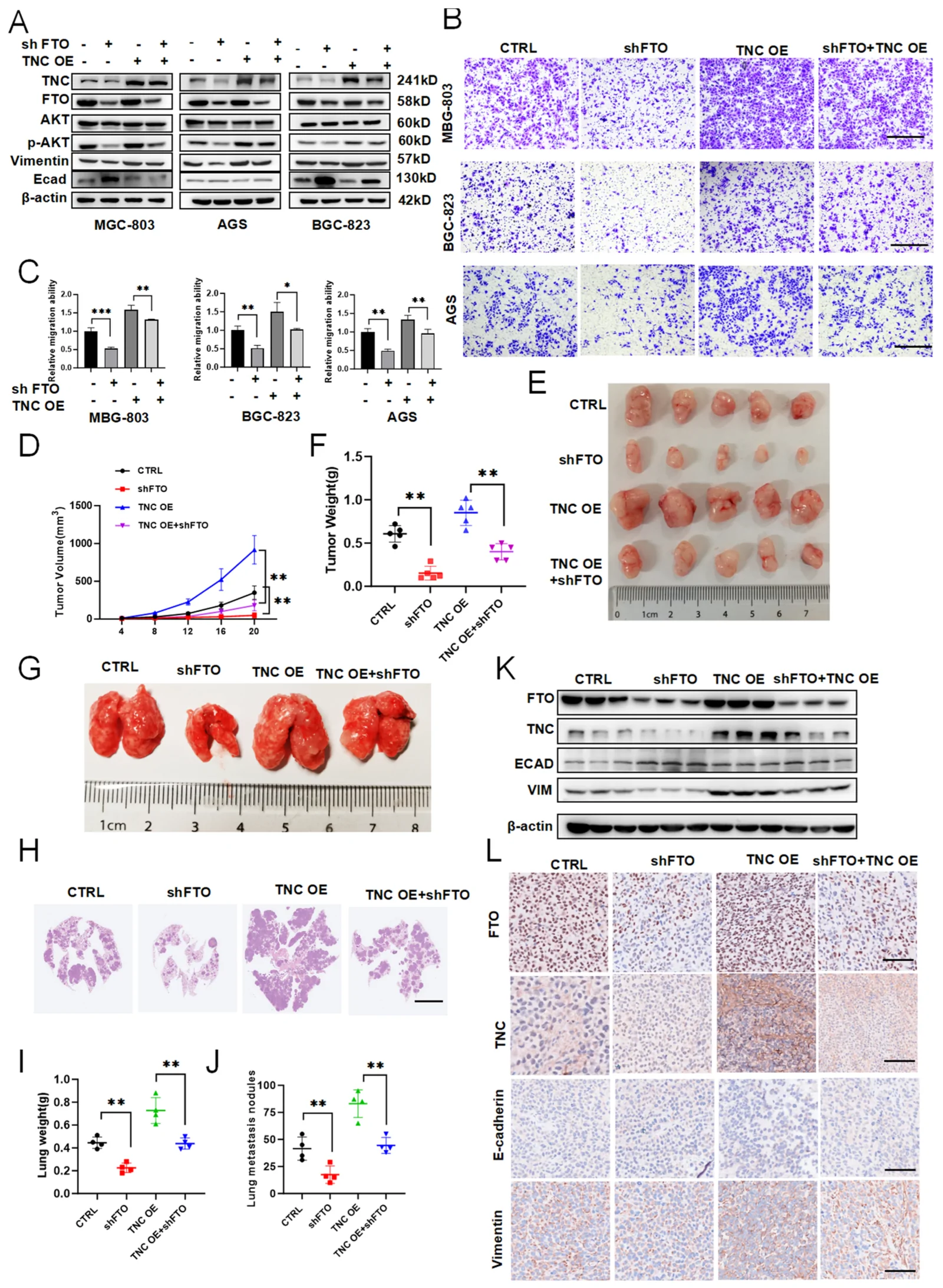
FTO-mediated epigenetic activation of TNC contributes to EMT and GC progression. (**A**) Western blot analysis of FTO, TNC, AKT, p-AKT, vimentin, and E-cadherin in AGS, BGC-823, and MGC-803 cells with control (CTRL), sh*FTO*, *TNC* overexpression (*TNC* OE), and sh*FTO* + *TNC* OE. *ACTB* as internal control. (**B**,**C**) Transwell invasion assays in AGS, BGC-823, and MGC-803 cells with control (CTRL), sh*FTO*, *TNC* overexpression (*TNC* OE), and sh*FTO* + *TNC* OE. Scale bar: 200 μm. Representative images shown in (**B**) and quantitative statistics in (**C**). Scale bar: 200 μm. (**D**–**F**) Nude mice were injected with MGC-803 cells (CTRL, sh*FTO*, *TNC* OE, or sh*FTO* + *TNC* OE) subcutaneously. Growth curves were plotted based on tumor volumes (**D**), representative images of tumors (**E**), and tumor weights (**F**) three weeks after implantation. (**G**,**H**) Representative images of lung metastasis (**G**) and H&E staining of lung lobes (**H**) from metastatic tumors. Scale bar: 5 mm. (**I**,**J**) Quantitative analysis of lung metastasis weights (**I**) and metastatic lung nodules (**J**). (**K**) Western blot analysis of FTO, TNC, vimentin, and E-cadherin in xenograft tumors. *ACTB* as internal control. (**L**) Immunohistochemical staining of FTO, TNC, vimentin, and E-cadherin in xenograft tumors. Scale bar: 50 μm. Data shown as mean ± SD. Statistical analysis performed using Student’s *t*-test. * *p* < 0.05; ** *p* < 0.01; *** *p* < 0.001.

To determine whether the *FTO*/*TNC* axis contributes to EMT and metastasis in GC, *TNC* was overexpressed in *FTO* knockdown cells (Supplementary Figure S7A). Overexpression of *TNC* restored EMT, characterized by increased vimentin and p-AKT, decreased E-cadherin levels (Figure 6A). In the cell invasion assay, the results showed that *FTO* knockdown inhibited cell invasion, while *TNC* overexpression promoted this process. In *FTO* knockdown cells, *TNC* overexpression could reverse this process (Figure 6B,C). In colony formation and wound healing assays, the results showed that FTO knockdown inhibited cell proliferation and migration, while *TNC* overexpression promoted these processes. In *FTO* knockdown cells, *TNC* overexpression could reverse these effects (Supplementary Figure S7B,C).

To further verify whether *TNC* overexpression can rescue the inhibitory effects of *FTO* knockdown on primary tumor growth and lung metastasis in vivo. We injected CTRL, sh*FTO*, *TNC* OE, and *TNC* OE + sh*FTO* MGC-803 cells subcutaneously into mice. Tumor growth curve analysis showed that tumor growth was significantly slowed in the sh*FTO* group, while *TNC* OE group significantly promoted tumor growth. The tumor growth rate in the sh*FTO* + *TNC* OE group was faster than that in the sh*FTO* group (Figure 6D). After 20 days, mice were sacrificed, and the xenografts were collected and weighed. Tumors formed by sh*FTO* + *TNC* OE cells were significantly larger and heavier in weight compared to sh*FTO* cells (Figure 6E,F). The results of the lung metastasis experiment showed that sh*FTO* + *TNC* OE cells generated more metastatic nodules and heavier tumors compared to the sh*FTO* cell (Figure 6G–J). Moreover, *TNC* overexpression restored vimentin expression and reduced E-cadherin levels in *FTO* knockdown tumors (Figure 6K,L). These findings confirm that *TNC* overexpression is responsible for GC’s FTO-mediated EMT and tumor progression.

## 4. Discussion

In this study, we uncovered an epigenetic mechanism that drives gastric cancer metastasis: the m6A demethylase FTO stabilizes *TNC* mRNA, leading to enhanced EMT and metastatic capability of GC cells. Through comprehensive analysis of 33 known m6A regulators, we identified FTO (Fat Mass and Obesity-Associated protein) as a key player in stabilizing *TNC* expression by reducing the m6A modification of *TNC* mRNA. Furthermore, we identified YTHDF2 as the critical m6A reader that recognizes the methylated *TNC* 3’UTR and promotes its decay. This stabilization enhances GC progression and, to our knowledge, this is the first study linking *TNC* to reader-dependent m6A-mediated regulation. Our findings highlight the therapeutic potential of targeting the *FTO*/*TNC*/EMT axis in treating gastric cancer.

Distant metastasis remains responsible for over 90% of cancer-related deaths, posing a significant challenge in cancer treatment [21]. TNC, an extracellular matrix (ECM) protein, is frequently overexpressed in various malignancies and is recognized for promoting metastasis [7,8,9,10]. In gastric cancer, *TNC* is notably upregulated and associated with poor prognosis [11]. However, the precise role of TNC in GC metastasis has been underexplored. Recent studies, such as that by Atsuko Yonemura et al., identified TNC as a key factor in peritoneal dissemination, highlighting its role in mesothelial–mesenchymal transition (MMT) and promoting peritoneal metastatic colonization [22].

Additionally, Xing Kang et al. reported that *TNC* knockdown reduced mesenteric metastatic nodules in a peritoneal cavity model [12]. Our in vivo results further support the role of TNC in GC metastasis, as GC cells with elevated *TNC* levels formed more lung metastasis nodules, suggesting that TNC facilitates both the extravasation of GC cells into distant organs and their colonization in the lung. Through bioinformatics analysis and gain-of-function (GOF) and loss-of-function (LOF) experiments, we found that *TNC* is strongly associated with EMT and plays a crucial role in GC cell proliferation, migration, and invasion. Given that EMT is central to cancer metastasis, therapy resistance, and tumor stemness [23], our findings suggest that targeting TNC-induced EMT could provide a promising strategy for controlling GC progression and metastasis.

Recent studies have highlighted the critical roles of extracellular matrix remodeling, tumor–stroma interactions, and EMT in gastric cancer progression [24]. TNC, as a matricellular protein, is not merely a passive component of the ECM but actively modulates cell–matrix interactions and promotes invasive phenotypes [10]. Our findings are consistent with these emerging concepts, demonstrating that FTO-mediated upregulation of *TNC* drives EMT and metastatic behavior. Moreover, the link between m6A modification and ECM remodeling adds a new layer of regulation to the complex network of tumor–stroma communication in GC.

At the genetic level, the *TNC* gene promoter contains a TATA box and is regulated by transcription factors such as *Prrx1*, *Evx1*, and *SOX4*, contributing to its expression patterns [10,14,15,16]. Epigenetically, *TNC* expression can be induced by JNK signaling and suppressed by metastasis-suppressing microRNAs such as *miR-335* [17,18]. Additionally, *TNC* protein stability is regulated by Lys63-ubiquitination via *Skp2*, promoting its recognition by *p62* and subsequent autophagic degradation [25]. This study identified a novel epigenetic regulatory mechanism of *TNC* expression through RNA m6A methylation. This process is mediated by FTO, which, by reducing m6A modifications, stabilizes *TNC* mRNA and promotes its expression, highlighting the role of FTO in regulating *TNC* in cancer contexts.

FTO, a key m6A demethylase, has been implicated as an oncogene in various cancers, including gastric [26,27,28,29]. While previous studies have primarily relied on bioinformatics analyses and in vitro models, our study strengthens these findings by incorporating clinical evaluations and both in vivo and in vitro experimental models. Our clinical assessments revealed frequent upregulation of FTO expression in GC tissues across two distinct cohorts. Furthermore, using an animal model derived from MGC-803 cells, we demonstrated that knockdown of *FTO* significantly inhibited tumor progression and reversed the EMT phenotype. These results reinforce the oncogenic role of FTO in GC.

m6A modification regulates gene expression by affecting RNA stability, promoting degradation or enhancing stability depending on the context [30]. Previous studies have suggested that FTO may either decrease m6A levels to promote target gene stability in some cancers, such as breast cancer and AML [31,32] or induce m6A-mediated degradation in others [28,33]. In our study, as a demethylase, FTO likely reduces m6A levels, thereby stabilizing *TNC* mRNA. Our RNA pull-down results specifically point to YTHDF2, not IGF2BPs, as the major binder of the *TNC* m6A site. YTHDF2 is a well-characterized m6A reader that recognizes m6A-modified transcripts and promotes their decay through recruitment of the CCR4-NOT deadenylase complex [34]. In line with this function, our actinomycin D experiments confirm that FTO loss accelerates *TNC* degradation in a YTHDF2-dependent manner. This aligns with the general paradigm that YTHDF2 recognizes m6A-marked transcripts and targets them for degradation, and that FTO counteracts this by removing the mark. Future studies should investigate the role of other m6A readers, such as other YTH domain family proteins [35], *HNRNPA2B1* [36], and *IGF2BP* [37], in FTO-mediated regulation of TNC mRNA, although our data suggest these proteins are not the primary binders.

We further demonstrate that the PI3K-AKT pathway is both necessary and sufficient for TNC-mediated EMT. Pharmacological activation of AKT rescues the migratory defect induced by *FTO* loss, whereas PI3K inhibition blocks this rescue. This is consistent with previous reports linking TNC to AKT signaling, but our study provides the first functional evidence that this pathway is the primary effector of the *FTO*/*TNC* axis in GC.

Recent reports on FTO-specific inhibitors, such as R-2-Hydroxyglutarate (**R-2HG**), **FB23**, and **CS1**, show potential in inhibiting FTO’s m6A demethylase activity in acute myeloid leukemia (AML) [38,39]. However, their efficacy in solid tumors like GC has yet to be fully evaluated, especially given the complex extracellular matrix of solid tumors that may hinder the penetration of targeted therapies. Notably, TNC is known to contribute to an immune-suppressive tumor microenvironment by influencing immune cell retention and activation [25,40,41]. Targeting FTO could reduce *TNC* expression, potentially creating an immune-liberating environment and enhancing the effectiveness of immune checkpoint therapies. Therefore, combining FTO-targeted therapy with immune checkpoint inhibitors could represent a promising approach for GC patients with high *FTO* and *TNC* expression.

From a clinical perspective, our findings suggest that the *FTO*/*TNC* axis may serve as a useful prognostic biomarker for gastric cancer. In our cohort, high expression of both *FTO* and *TNC* was associated with significantly poorer overall survival, supporting their potential as predictive indicators of aggressive disease. Furthermore, targeting this axis offers a promising therapeutic avenue. FTO-specific inhibitors, such as **R-2HG**, and **FB23**, have shown efficacy in preclinical models of AML [38,39], and their application in solid tumors like GC warrants investigation. Given that TNC contributes to an immune-suppressive tumor microenvironment [42], combining FTO-targeted therapy with immune checkpoint blockade could potentially enhance antitumor immunity and improve clinical outcomes in GC patients with elevated *FTO*/*TNC* expression.

However, our study also has some limitations. One limitation of our study is the lack of in vivo testing of pharmacological FTO inhibition, whether this approach can effectively reduce metastasis in GC models requires further validation. Additionally, although we identified site-3 in the 3’UTR of *TNC* as a critical m6A site, the contribution of other predicted sites and potential compensatory mechanisms remain to be explored. Additionally, the publicly available TCGA-STAD and GEO datasets used in this study may have inherent batch effects and cohort heterogeneity; our findings should be validated in independent prospective cohorts.

Strengths of this study include the combination of multi-omics correlation analysis, rigorous in vivo models (subcutaneous and tail-vein metastasis), mechanistic dissection of m6A reader function, and pathway rescue experiments. These data collectively provide a comprehensive understanding of the *FTO*/*TNC*/EMT axis.

In conclusion, this work identifies a previously unrecognized *FTO*/*TNC*/EMT axis driving GC metastasis, with YTHDF2 as the key reader and PI3K-AKT as the downstream effector. However, it is important to acknowledge that the translational implications of these findings remain largely preclinical. The lack of in vivo pharmacological FTO inhibition and the need for validation in independent patient cohorts are notable limitations. Nonetheless, our results suggest that targeting this axis may offer a promising strategy for curbing metastasis, particularly in GC patients with elevated *FTO* and *TNC* expression, pending further translational studies.

## Figures and Tables

**Table 1:** Univariate and multivariate Cox regression of overall survival for gastric cancer patients.

Characteristics	Univariate			Multivariate		
	HR	95% CI	*p*	HR	95% CI	*p*
Age, y						
<60	1	0.716–2.229	0.403			
>60	1.283					
Gender						
Male	1	0.687–1.935	0.591			
Female	1.153					
Tumor differentiation						
Well/Moderate	1	0.580–1.915	0.865			
Poor	1.053					
Stage						
I–II	1	1.939–6.308	<0.001	1	1.449–5.110	0.002
III–IV	3.498			2.722		
Venous invasion						
No	1	1.458–4.113	0.001	1	0.788–2.818	0.220
Yes	2.449			1.490		
Nervous invasion						
No	1	1.153–3.987	0.016	1	0.533–2.344	0.769
Yes	2.144			1.117		
TNC expression						
Low	1	1.296–3.721	0.003	1	1.008–2.982	0.047
High	2.196			1.734		

CI, confidence interval; HR, hazard ratio. Univariate *p* values were derived from log-rank test. Variables with *p* < 0.05 in univariate analysis (AJCC stage, venous invasion, nervous invasion, and TNC expression) were entered into multivariate Cox regression analysis. Multivariate *p* values were derived from Cox regression analysis including these four variables.

## Data Availability

The data supporting the findings of this study are available from the corresponding author upon reasonable request.

## Supplementary Materials

The following supporting information can be downloaded at https://www.mdpi.com/article/10.3390/biomedicines14091958/s1. Figure S1: TNC knockdown inhibited the proliferation, invasion, and migration of GC cells without influencing apoptosis; Figure S2: TNC overexpression promoted proliferation invasion and migration of GC cells; Figure S3: TNC knockdown inhibited EMT in vivo; Figure S4: PI3K-AKT activation rescued the migratory and invasive defects induced by FTO knockdown in GC cells; Figure S5: FTO knockdown inhibited the proliferation, invasion, and migration of GC cells without influencing apoptosis; Figure S6: FTO knockdown inhibited EMT of GC cells; Figure S7: FTO-mediated decrease in TNC expression inhibited the proliferation and migration of GC cells; Table S1: Antibodies, critical commercial assays, and sequence of the primers; Table S2: Clinicopathological information for gastric cancer patients.

## References

[1] Bray F., Laversanne M., Sung H., Ferlay J., Siegel R.L., Soerjomataram I., Jemal A. (2024). Global cancer statistics 2022: GLOBOCAN estimates of incidence and mortality worldwide for 36 cancers in 185 countries. CA Cancer J. Clin..

[2] Liu K.S., Raza S.A., El-Serag H.B., Thrift A.P. (2024). Recent Trends in the Incidence of Gastric Cancer in the United States. J. Clin. Gastroenterol..

[3] Tan M.C., Sen A., Kligman E., Othman M.O., Liu Y., El-Serag H.B., Thrift A.P. (2023). Validation of a pre-endoscopy risk score for predicting the presence of gastric intestinal metaplasia in a U.S. population. Gastrointest. Endosc..

[4] Zheng R.S., Chen R., Han B.F., Wang S.M., Li L., Sun K.X., Zeng H.M., Wei W.W., He J. (2024). Cancer incidence and mortality in China, 2022. Zhonghua Zhong Liu Za Zhi.

[5] Satala C.B., Jung I., Gurzu S. (2023). Mucin-Phenotype and Expression of the Protein V-Set and Immunoglobulin Domain Containing 1 (*VSIG1*): New Insights into Gastric Carcinogenesis. Int. J. Mol. Sci..

[6] Lei Z.N., Teng Q.X., Tian Q., Chen W., Xie Y., Wu K., Zeng Q., Zeng L., Pan Y., Chen Z.S. (2022). Signaling pathways and therapeutic interventions in gastric cancer. Signal Transduct. Target. Ther..

[7] Oskarsson T., Acharyya S., Zhang X.H., Vanharanta S., Tavazoie S.F., Morris P.G., Downey R.J., Manova-Todorova K., Brogi E., Massagué J. (2011). Breast cancer cells produce tenascin C as a metastatic niche component to colonize the lungs. Nat. Med..

[8] Hongu T., Pein M., Insua-Rodríguez J., Gutjahr E., Mattavelli G., Meier J., Decker K., Descot A., Bozza M., Harbottle R. (2022). Perivascular tenascin C triggers sequential activation of macrophages and endothelial cells to generate a pro-metastatic vascular niche in the lungs. Nat. Cancer.

[9] Sun Z., Velázquez-Quesada I., Murdamoothoo D., Ahowesso C., Yilmaz A., Spenlé C., Averous G., Erne W., Oberndorfer F., Oszwald A. (2019). Tenascin-C increases lung metastasis by impacting blood vessel invasions. Matrix Biol..

[10] Midwood K.S., Chiquet M., Tucker R.P., Orend G. (2016). Tenascin-C at a glance. J. Cell Sci..

[11] Qi W., Yang Z., Li H., Cui Y., Xuan Y. (2019). The role of Tenascin-C and *Twist1* in gastric cancer: Cancer progression and prognosis. Apmis.

[12] Kang X., Xu E., Wang X., Qian L., Yang Z., Yu H., Wang C., Ren C., Wang Y., Lu X. (2021). Tenascin-c knockdown suppresses vasculogenic mimicry of gastric cancer by inhibiting ERK- triggered EMT. Cell Death Dis..

[13] Chiovaro F., Chiquet-Ehrismann R., Chiquet M. (2015). Transcriptional regulation of tenascin genes. Cell Adh. Migr..

[14] Fischer D., Chiquet-Ehrismann R., Bernasconi C., Chiquet M. (1995). A single heparin binding region within the fibrinogen-like domain is functional in chick tenascin-C. J. Biol. Chem..

[15] Jones F.S., Chalepakis G., Gruss P., Edelman G.M. (1992). Activation of the cytotactin promoter by the homeobox-containing gene *Evx-1*. Proc. Natl. Acad. Sci. USA.

[16] Scharer C.D., McCabe C.D., Ali-Seyed M., Berger M.F., Bulyk M.L., Moreno C.S. (2009). Genome-wide promoter analysis of the *SOX4* transcriptional network in prostate cancer cells. Cancer Res..

[17] Insua-Rodríguez J., Pein M., Hongu T., Meier J., Descot A., Lowy C.M., De Braekeleer E., Sinn H.P., Spaich S., Sütterlin M. (2018). Stress signaling in breast cancer cells induces matrix components that promote chemoresistant metastasis. EMBO Mol. Med..

[18] Graham É.A., Mallet J.F., Jambi M., Nishioka H., Homma K., Matar C. (2017). MicroRNA signature in the chemoprevention of functionally-enriched stem and progenitor pools (FESPP) by Active Hexose Correlated Compound (AHCC). Cancer Biol. Ther..

[19] Fu J., Cui X., Zhang X., Cheng M., Li X., Guo Z., Cui X. (2021). The Role of m6A Ribonucleic Acid Modification in the Occurrence of Atherosclerosis. Front. Genet..

[20] Zhang H., Shi X., Huang T., Zhao X., Chen W., Gu N., Zhang R. (2020). Dynamic landscape and evolution of m6A methylation in human. Nucleic Acids Res..

[21] Gerstberger S., Jiang Q., Ganesh K. (2023). Metastasis. Cell.

[22] Yonemura A., Semba T., Zhang J., Fan Y., Yasuda-Yoshihara N., Wang H., Uchihara T., Yasuda T., Nishimura A., Fu L. (2024). Mesothelial cells with mesenchymal features enhance peritoneal dissemination by forming a protumorigenic microenvironment. Cell Rep..

[23] Lengrand J., Pastushenko I., Vanuytven S., Song Y., Venet D., Sarate R.M., Bellina M., Moers V., Boinet A., Sifrim A. (2023). Pharmacological targeting of netrin-1 inhibits EMT in cancer. Nature.

[24] Satala C.B., Gurau G., Gurau A.M., Patrichi G., Mihalache D. (2026). Tumor Budding in Gastric Carcinoma: Beyond Counting Cells at the Invasive Front-A Review of Current Evidence and Biological Perspectives. Int. J. Mol. Sci..

[25] Li Z.L., Zhang H.L., Huang Y., Huang J.H., Sun P., Zhou N.N., Chen Y.H., Mai J., Wang Y., Yu Y. (2020). Autophagy deficiency promotes triple-negative breast cancer resistance to T cell-mediated cytotoxicity by blocking tenascin-C degradation. Nat. Commun..

[26] Xu D., Shao W., Jiang Y., Wang X., Liu Y., Liu X. (2017). *FTO* expression is associated with the occurrence of gastric cancer and prognosis. Oncol. Rep..

[27] Li Y., Xiao J., Bai J., Tian Y., Qu Y., Chen X., Wang Q., Li X., Zhang Y., Xu J. (2019). Molecular characterization and clinical relevance of m(6)A regulators across 33 cancer types. Mol. Cancer.

[28] Wang D., Qu X., Lu W., Wang Y., Jin Y., Hou K., Yang B., Li C., Qi J., Xiao J. (2021). N(6)-Methyladenosine RNA Demethylase *FTO* Promotes Gastric Cancer Metastasis by Down-Regulating the m6A Methylation of *ITGB1*. Front. Oncol..

[29] Shimura T., Kandimalla R., Okugawa Y., Ohi M., Toiyama Y., He C., Goel A. (2022). Novel evidence for m(6)A methylation regulators as prognostic biomarkers and *FTO* as a potential therapeutic target in gastric cancer. Br. J. Cancer.

[30] Zhou Z., Lv J., Yu H., Han J., Yang X., Feng D., Wu Q., Yuan B., Lu Q., Yang H. (2020). Mechanism of RNA modification N6-methyladenosine in human cancer. Mol. Cancer.

[31] Li Z., Weng H., Su R., Weng X., Zuo Z., Li C., Huang H., Nachtergaele S., Dong L., Hu C. (2017). *FTO* Plays an Oncogenic Role in Acute Myeloid Leukemia as a N(6)-Methyladenosine RNA Demethylase. Cancer Cell.

[32] Niu Y., Lin Z., Wan A., Chen H., Liang H., Sun L., Wang Y., Li X., Xiong X.F., Wei B. (2019). RNA N6-methyladenosine demethylase *FTO* promotes breast tumor progression through inhibiting *BNIP3*. Mol. Cancer.

[33] Tan Z., Shi S., Xu J., Liu X., Lei Y., Zhang B., Hua J., Meng Q., Wang W., Yu X. (2022). RNA N6-methyladenosine demethylase *FTO* promotes pancreatic cancer progression by inducing the autocrine activity of *PDGFC* in an m(6)A-*YTHDF2*-dependent manner. Oncogene.

[34] Zaccara S., Jaffrey S.R. (2024). Understanding the redundant functions of the m(6)A-binding YTHDF proteins. RNA.

[35] Zaccara S., Ries R.J., Jaffrey S.R. (2019). Reading, writing and erasing mRNA methylation. Nat. Rev. Mol. Cell Biol..

[36] Alarcón C.R., Goodarzi H., Lee H., Liu X., Tavazoie S., Tavazoie S.F. (2015). *HNRNPA2B1* Is a Mediator of m(6)A-Dependent Nuclear RNA Processing Events. Cell.

[37] Huang H., Weng H., Sun W., Qin X., Shi H., Wu H., Zhao B.S., Mesquita A., Liu C., Yuan C.L. (2018). Recognition of RNA N(6)-methyladenosine by *IGF2BP* proteins enhances mRNA stability and translation. Nat. Cell Biol..

[38] Su R., Dong L., Li C., Nachtergaele S., Wunderlich M., Qing Y., Deng X., Wang Y., Weng X., Hu C. (2018). **R-2HG** Exhibits Anti-tumor Activity by Targeting *FTO*/m(6)A/*MYC*/*CEBPA* Signaling. Cell.

[39] Huang Y., Su R., Sheng Y., Dong L., Dong Z., Xu H., Ni T., Zhang Z.S., Zhang T., Li C. (2019). Small-Molecule Targeting of Oncogenic FTO Demethylase in Acute Myeloid Leukemia. Cancer Cell.

[40] Murdamoothoo D., Sun Z., Yilmaz A., Riegel G., Abou-Faycal C., Deligne C., Velazquez-Quesada I., Erne W., Nascimento M., Mörgelin M. (2021). Tenascin-C immobilizes infiltrating T lymphocytes through *CXCL12* promoting breast cancer progression. EMBO Mol. Med..

[41] Spenlé C., Loustau T., Murdamoothoo D., Erne W., Beghelli-de la Forest Divonne S., Veber R., Petti L., Bourdely P., Mörgelin M., Brauchle E.M. (2020). Tenascin-C Orchestrates an Immune-Suppressive Tumor Microenvironment in Oral Squamous Cell Carcinoma. Cancer Immunol. Res..

[42] Wang Y., Wen X., Su C., You Y., Jiang Z., Fan Q., Zhu D. (2025). The role of tenascin-C in tumor microenvironments and its potential as a therapeutic target. Front. Cell Dev. Biol..

