# Supplementary material for: FTO-Driven m6A Demethylation of TNC Promotes Epithelial–Mesenchymal Transition and Gastric Cancer Metastasis
Source: Biomedicines. 2026 Aug 31;14(9):1958. doi: 10.3390/biomedicines14091958 (PMC13604668; doi:10.3390/biomedicines14091958)
Supplement: Supplementary file 1 [file biomedicines-14-01958-s001.zip › Sup Figures.pdf]

Figure S1

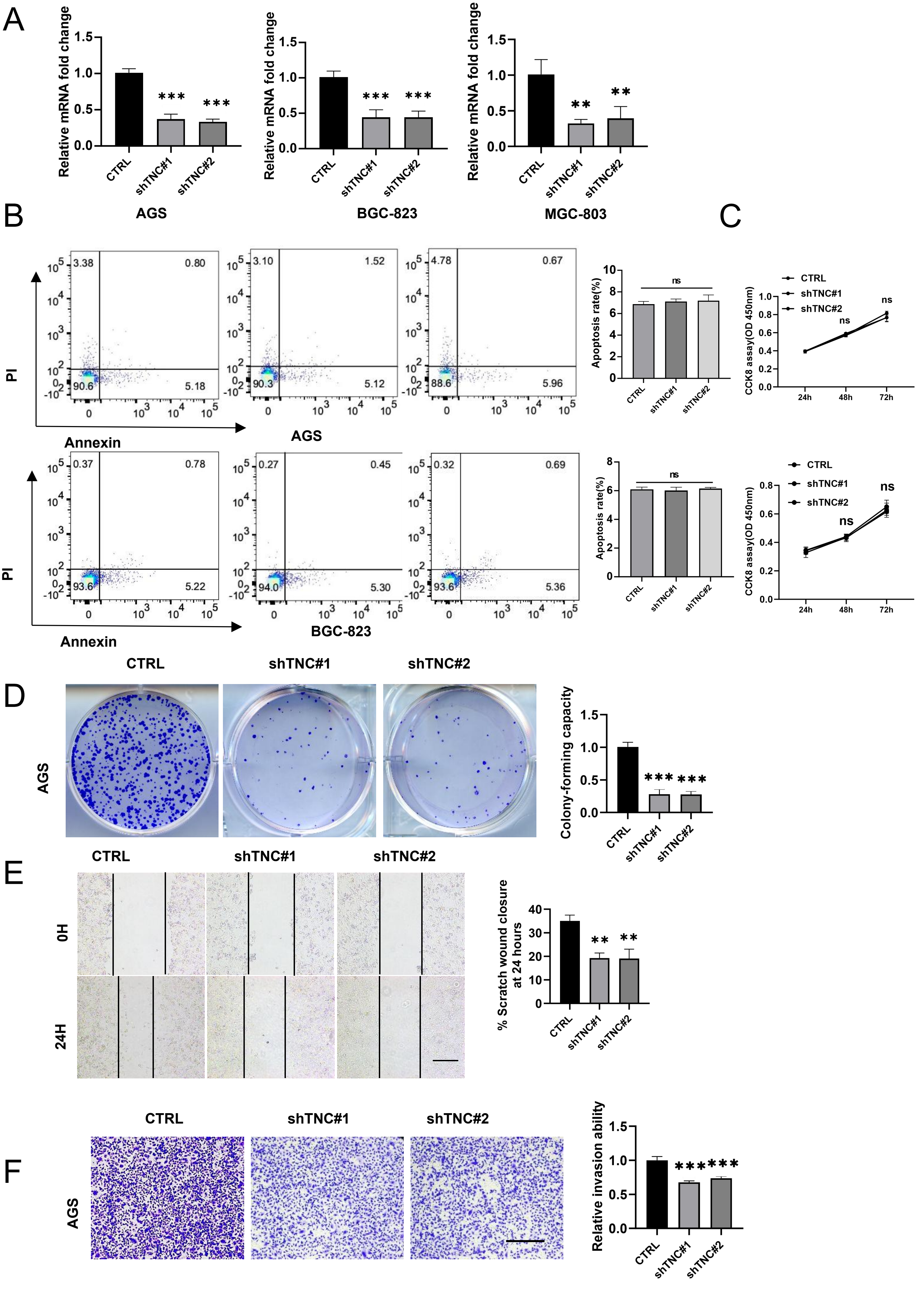
A
Relative mRNA fold change
1.5
1.0
0.5
0.0
***
**
CTRL
shTNC#1
shTNC#2
AGS
BGC-823
MGC-803
B
C
PI
Annexin
3.38
0.80
90.6
5.18
3.10
1.52
90.3
5.12
4.78
0.67
88.6
5.96
AGS
0.37
0.78
93.6
5.22
0.27
0.45
94.0
5.30
0.32
0.69
93.6
5.36
BGC-823
Apoptosis rate(%)
ns
CCK8 assay(OD 450nm)
24h
48h
72h
D
AGS
Colony-forming capacity
E
0H
24H
% Scratch wound closure at 24 hours
F
Relative invasion ability

Figure S2

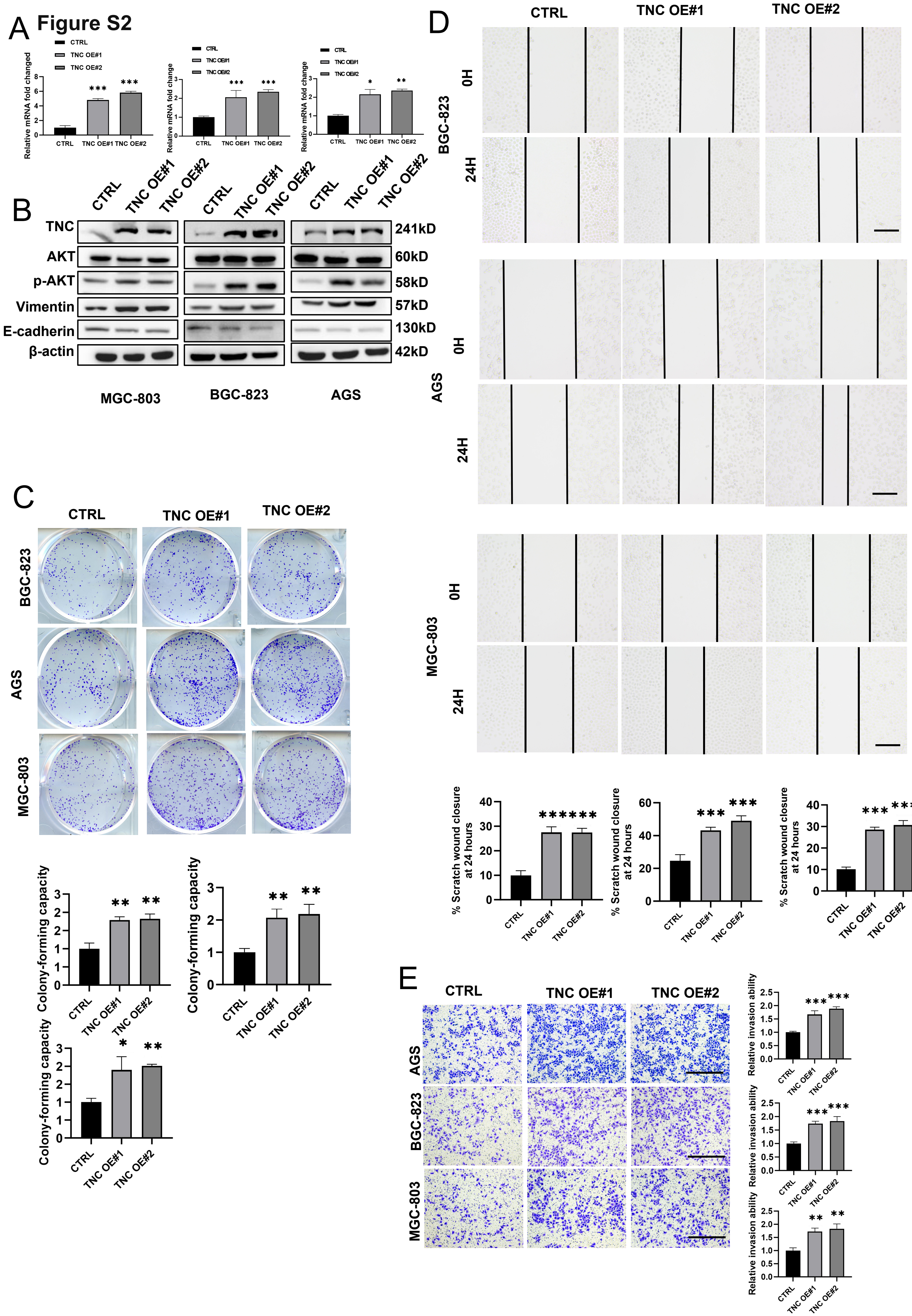

A
CTRL
TNC OE#1
TNC OE#2
Relative mRNA fold changed
Relative mRNA fold change
B
TNC
AKT
p-AKT
Vimentin
E-cadherin
β-actin
241kD
60kD
58kD
57kD
130kD
42kD
MGC-803
BGC-823
AGS
C
Colony-forming capacity
D
0H
24H
% Scratch wound closure at 24 hours
E
Relative invasion ability

Figure S3

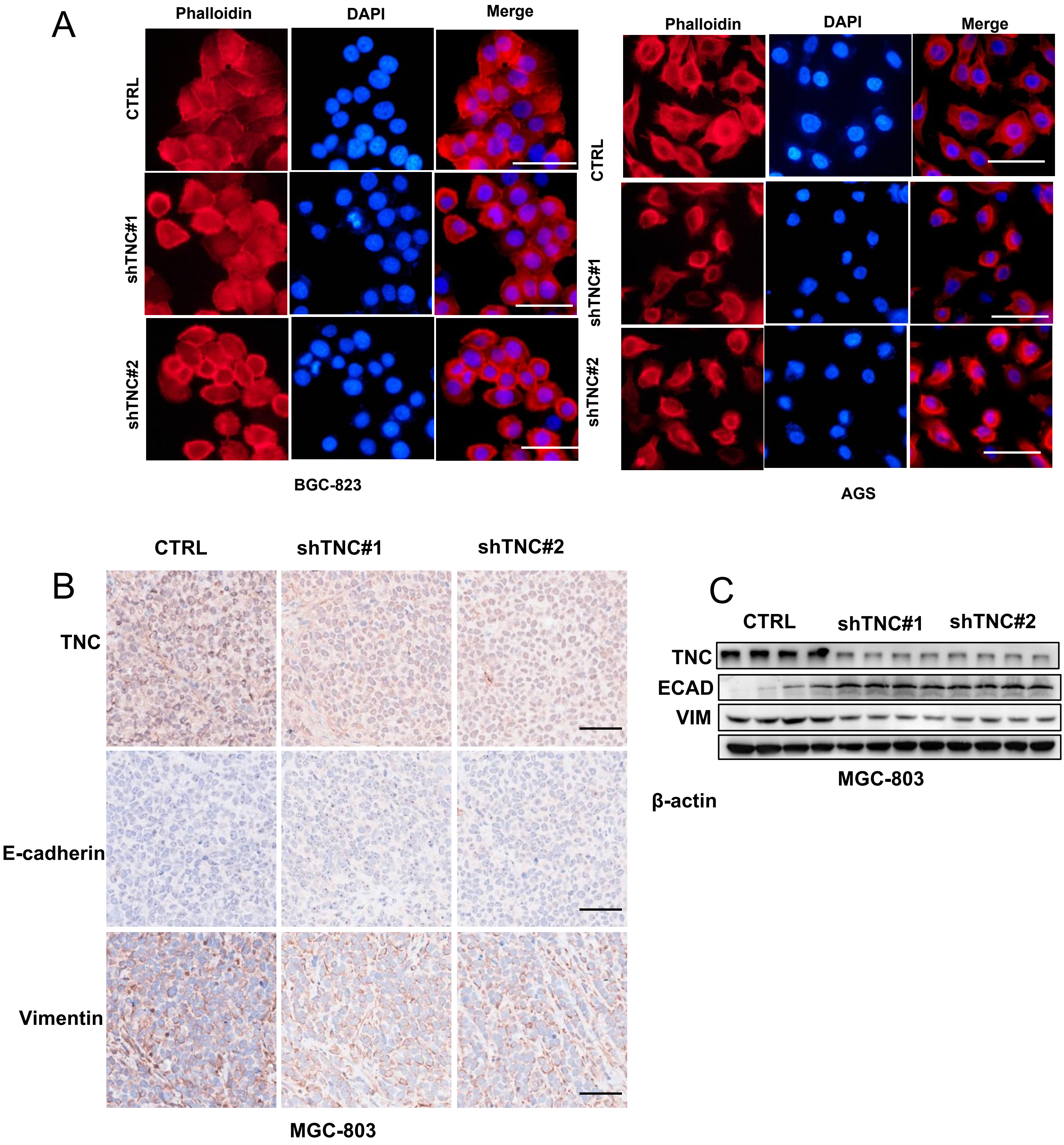

A
Phalloidin
DAPI
Merge
CTRL
shTNC#1
shTNC#2
BGC-823
Phalloidin
DAPI
Merge
CTRL
shTNC#1
shTNC#2
AGS
B
CTRL
shTNC#1
shTNC#2
TNC
E-cadherin
Vimentin
MGC-803
C
CTRL
shTNC#1
shTNC#2
TNC
ECAD
VIM
β-actin
MGC-803

Figure S4

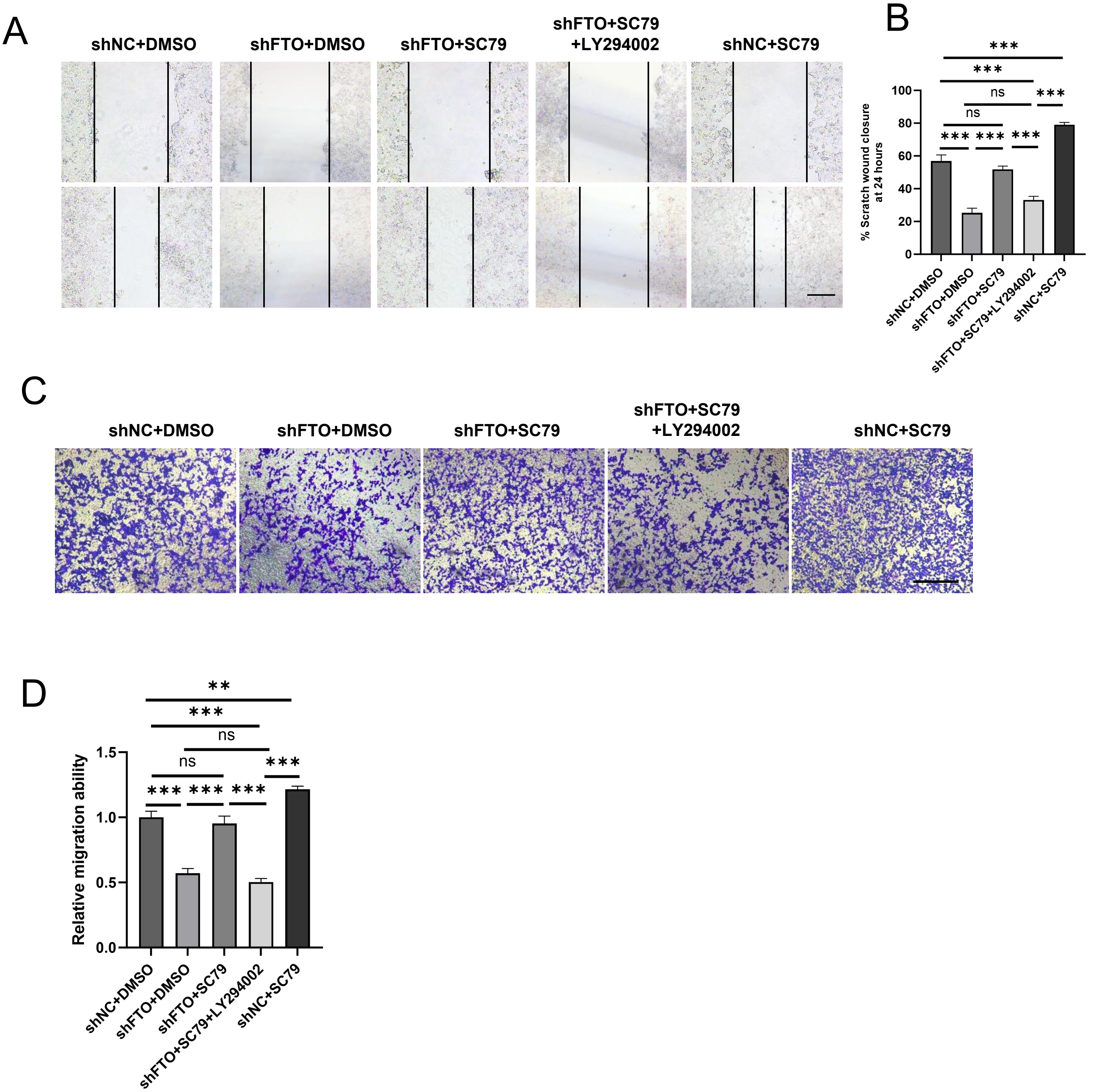
A
shNC+DMSO
shFTO+DMSO
shFTO+SC79
shFTO+SC79
+LY294002
shNC+SC79
B
% Scratch wound closure
at 24 hours
100
80
60
40
20
0
***
***
ns
***
ns
*** *** ***
shNC+DMSO
shFTO+DMSO
shFTO+SC79
shFTO+SC79+LY294002
shNC+SC79
C
shNC+DMSO
shFTO+DMSO
shFTO+SC79
shFTO+SC79
+LY294002
shNC+SC79
D
Relative migration ability
1.5
1.0
0.5
0.0
**
***
ns
ns
***
*** *** ***
shNC+DMSO
shFTO+DMSO
shFTO+SC79
shFTO+SC79+LY294002
shNC+SC79

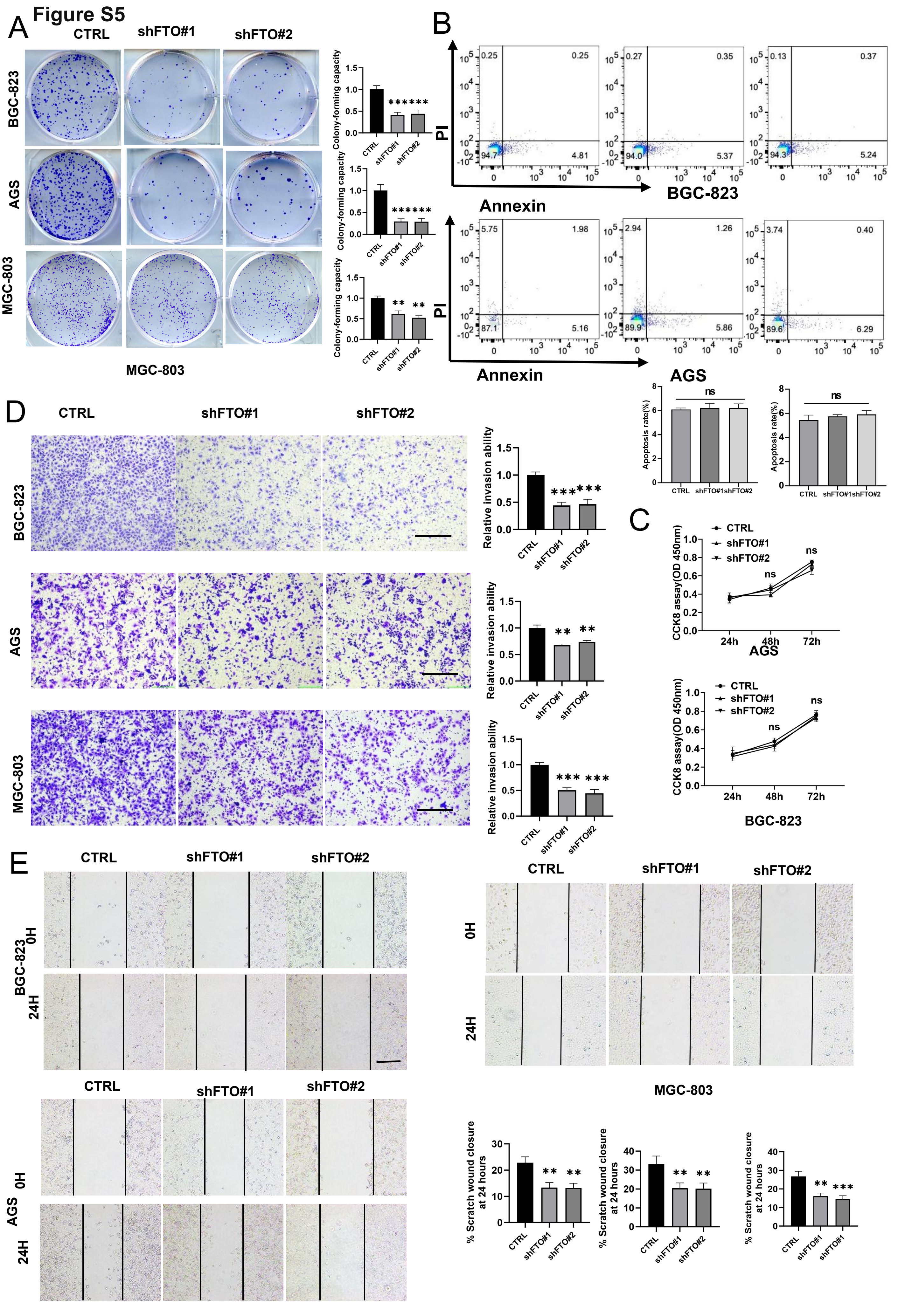

Figure S5
A
CTRL
shFTO#1
shFTO#2
BGC-823
AGS
MGC-803
Colony-forming capacity
CTRL
shFTO#1
shFTO#2
MGC-803
B
PI
Annexin
BGC-823
AGS
Apoptosis rate(%)
ns
C
CTRL
shFTO#1
shFTO#2
CCK8 assay(OD 450nm)
24h
48h
72h
AGS
BGC-823
D
Relative invasion ability
E
0H
24H
MGC-803
% Scratch wound closure at 24 hours

Figure S6

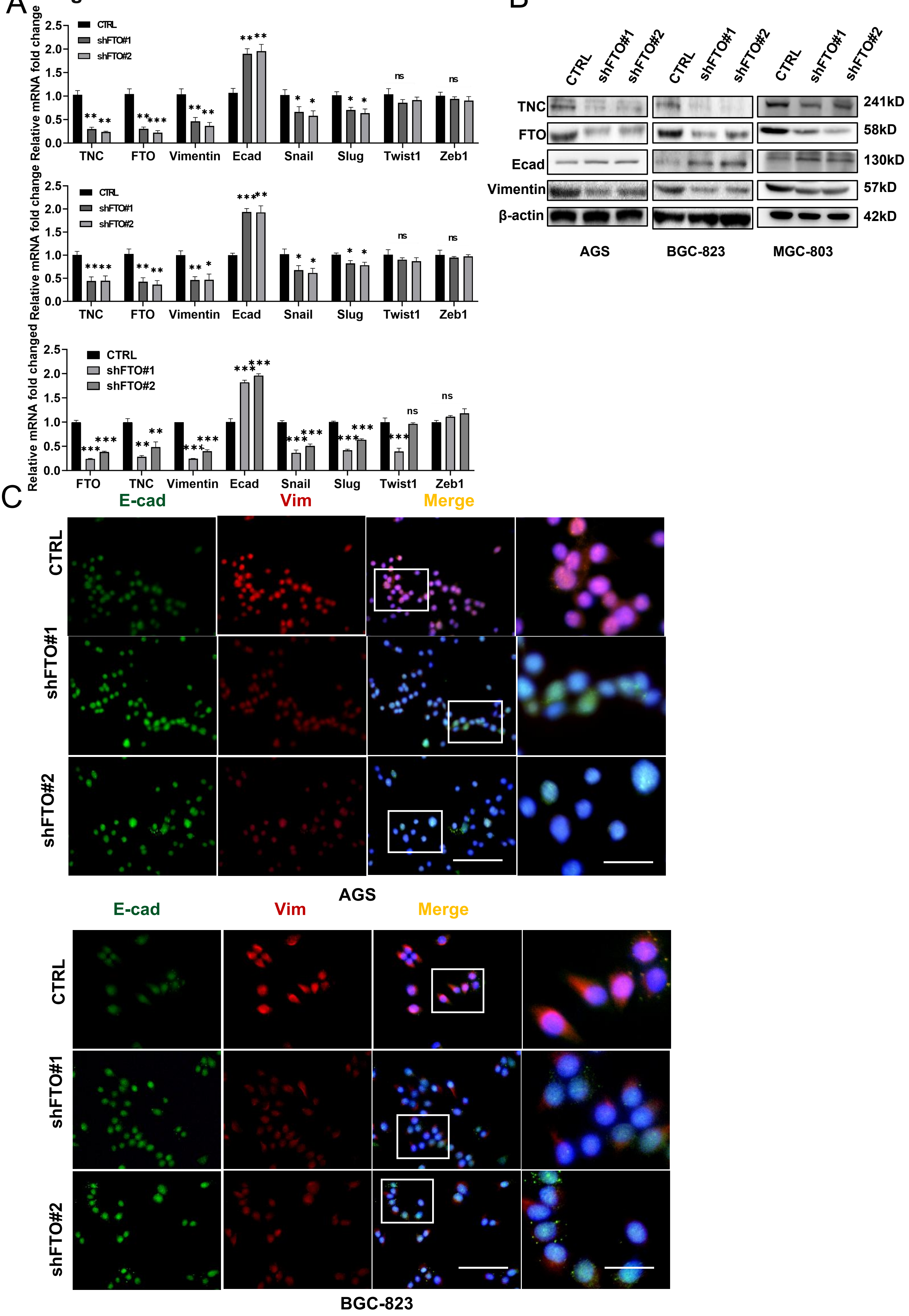
A
Relative mRNA fold change
CTRL
shFTO#1
shFTO#2
TNC
FTO
Vimentin
Ecad
Snail
Slug
Twist1
Zeb1
ns
Relative mRNA fold changed
B
TNC
FTO
Ecad
Vimentin
β-actin
241kD
58kD
130kD
57kD
42kD
AGS
BGC-823
MGC-803
C
E-cad
Vim
Merge
AGS
BGC-823

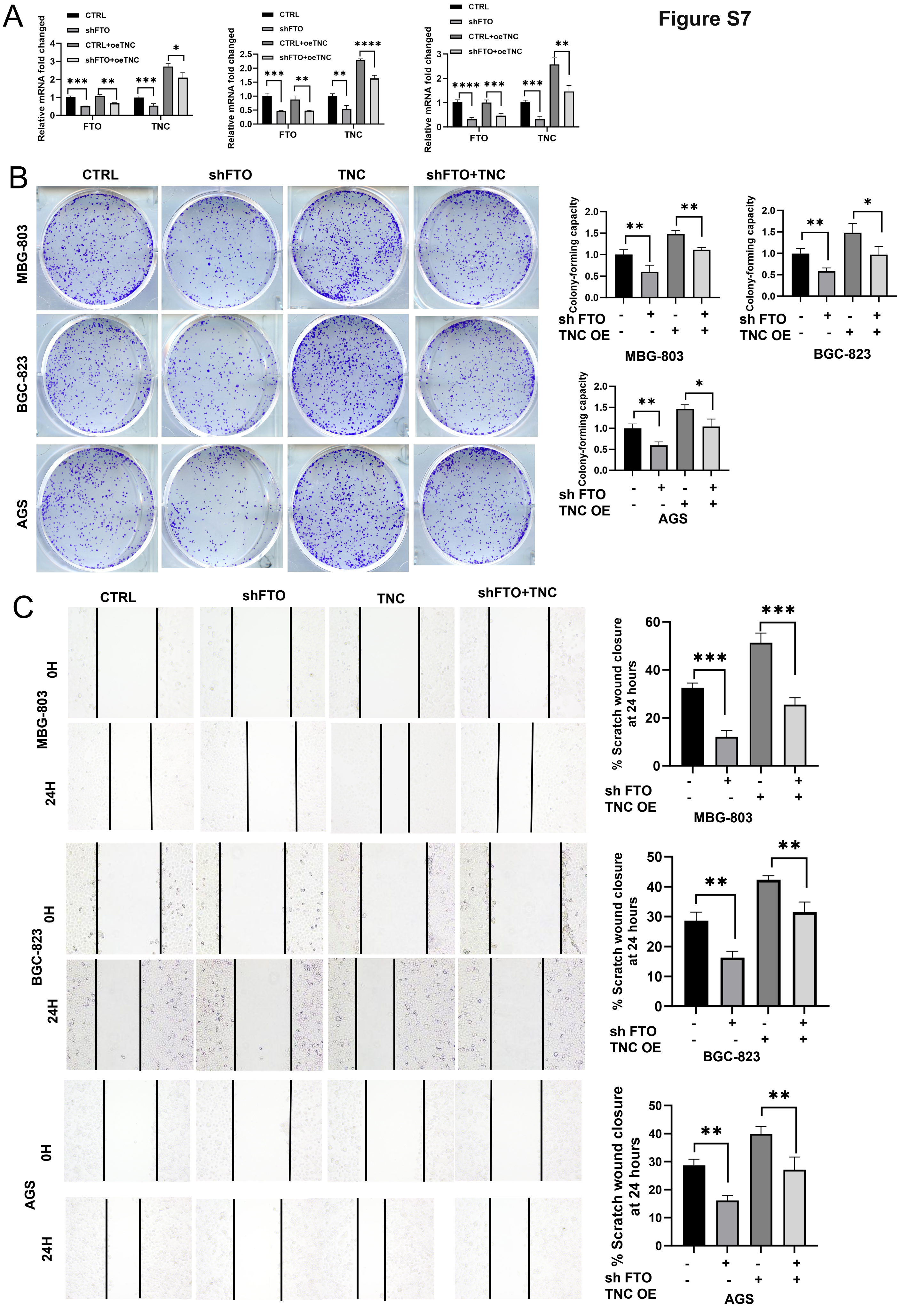

Figure S7
A
CTRL
shFTO
CTRL+oeTNC
shFTO+oeTNC
Relative mRNA fold changed
FTO
TNC
B
CTRL
shFTO
TNC
shFTO+TNC
MBG-803
BGC-823
AGS
Colony-forming capacity
sh FTO
TNC OE
C
0H
24H
% Scratch wound closure at 24 hours

Figure S1 TNC knockdown inhibited the proliferation, invasion, and migration of GC cells without influencing apoptosis.

(A) mRNA expression levels of TNC by CTRL or shTNC in AGS, BGC-823 and MGC-803 cells. Internal control was performed with ACTB. (B) The apoptosis assay was performed on AGS and BGC-823 cells with control (CTRL) or shTNC. Apoptosis was induced by treating cells with 10 g/mL 5-FU for 24 hours. Three biological replicates were included. Xaxis: annexin-V-FITC; Y-axis: PI. (C) Cell viability was assayed in CTRL and shTNC in AGS, BGC-823 cells. Three biological replicates were included. (D) The clone formation assay was performed with AGS cells with control (CTRL) or shTNC. Images shown were representative results of three biological replicates. (E) The wound healing assay was conducted using AGS cells with control (CTRL) or shTNC. Images shown were representative results of three biological replicates. Scale bar: 100 µm.(F) The transwell invasion assay was conducted using AGS cells with control (CTRL) or shTNC .Images shown were representative results of three biological replicates. Scale bar: 200µm. The data shown represented the mean ± SD. Statistical analyses were performed with the Student's t test. *$p < 0.05$; **$p < 0.01$; ***$p < 0.001$; ns stands for no significant change.

Figure S2 TNC overexpression promoted proliferation invasion and migration of GC cells.

(A) mRNA expression levels of TNC and FTO in AGS, BGC-823 and MGC-803 cells with control (CTRL) and TNC overexpression (TNC OE), Internal control was performed with ACTB. (B) Protein concentrations of FTO, TNC, Ecad, Vimentin, AKT, and p-AKT were measured by Western blot in AGS, BGC-823 and MGC-803 control (CTRL) and TNC OE cells. Internal control was performed with ACTB. (C) The clone formation assay was performed with AGS, BGC-823 and MGC-803 cells with control (CTRL) and TNC OE. Images shown were representative results of three

biological replicates. (D)The wound healing assay was conducted using AGS, BGC-823 and MGC-803 cells with control (CTRL)and TNC OE. Images shown were representative results of three biological replicates. Scale bar: 100 μm. (E)The transwell invasion assay was conducted using AGS, BGC-823 and MGC-803 cells with control (CTRL) or TNC OE. Images shown were representative results of three biological replicates. Scale bar: 200μm. The data shown represented the mean ± SD. Statistical analyses were performed with the Student's t test. *$p < 0.05$; **$p < 0.01$; ***$p < 0.001$; ns stands for no significant change.

Figure S3 TNC knockdown inhibited EMT in vivo.

(A) Representative IF staining images of Phalloidin(red) in AGS, BGC-823 cells with control (CTRL) or shFTO. Nuclei were stained in blue. Scale bar: 50 μm. (B) Using immunohistochemistry, TNC, Vimentin, and E-cadherin expression was detected in the tumors. Representative images were shown. Scale bar: 50 μm.(C) Western blot results of xenografts homogenates, TNC, Vimentin, and E-cadherin were probed. Internal control was performed with ACTB.

Figure S4 PI3K-AKT activation rescued the migratory and invasive defects induced by FTO knockdown in GC cells.

(A-B) The wound healing assay was conducted using AGS cells treated with DMSO (vehicle), SC79 (AKT activator), or SC79 + LY294002 (PI3K inhibitor) under shNC or shFTO background at 0 h and 24 h. Representative images shown in (A) and quantitative statistics in (B). Scale bar: 100 μm. (C-D) The transwell migration assay was conducted in AGS cells with the same treatment groups. Representative images shown in (C) and quantitative statistics in (D). Scale bar: 200 μm. Data are shown as

mean ± SD (n = 3 biological replicates). Statistical analyses were performed with the Student's t test. *$p < 0.05$, **$p < 0.01$, ***$p < 0.001$; ns, not significant.

Figure S5 FTO knockdown inhibited the proliferation, invasion, and migration of GC cells without influencing apoptosis.

(A) The clone formation assay was performed with AGS, BGC-823 and MGC-803 cells with control (CTRL) or shFTO. Images shown were representative results of three biological replicates. (B) The apoptosis assay was performed on AGS, BGC-823 cells with control (CTRL) or shFTO. Apoptosis was induced by treating cells with 10 g/mL 5-FU for 24 hours. Three biological replicates were included. Xaxis: annexin-V-FITC; Y-axis: PI. (C) Cell viability was assayed in CTRL and shFTO in AGS, BGC-823 cells. Three biological replicates are included. (D) The transwell invasion assay was conducted using AGS, BGC-823 and MGC-803 cells with control (CTRL) or shFTO. Images shown were representative results of three biological replicates. Scale bar: 200μm. (E) The wound healing assay was conducted using AGS, BGC-823 and MGC-803 cells with control (CTRL) or shFTO. Images shown were representative results of three biological replicates. Scale bar: 100 μm. The data shown represented the mean ± SD. Statistical analyses were performed with the Student's t test. *$p < 0.05$; **$p < 0.01$; ***$p < 0.001$; ns stands for no significant change.

Figure S6 FTO knockdown inhibited EMT of GC cells

(A) mRNA expression levels of FTO, TNC, Vimentin, Ecad, Snil, and Slug, Twist1, and Zeb1 in AGS, BGC-823 and MGC-803 control (CTRL) and shFTO cells. Internal control was performed with ACTB. (B) Protein concentrations of FTO, TNC, Ecad, and Vimentin were measured by Western blot in AGS, BGC-823 and MGC-803 control (CTRL) and shFTO cells.Internal control was performed with ACTB. (C) Images of immunofluorescence staining of E-cad (green) and Vim (red) in AGS, BGC-823 control (CTRL) and shFTO cells. Nuclei were stained in blue; Scale bar: 50 μm. Images shown

were representative results of three biological replicates. The data shown represented the mean ± SD. Statistical analyses were performed with the Student's t test. *$p < 0.05$; **$p < 0.01$; ***$p < 0.001$.

Figure S7 FTO-mediated decrease in TNC expression inhibited the proliferation and migration of GC cells.

(A) mRNA expression levels of FTO, TNC in AGS, BGC-823 and MGC-803 cells with control (CTRL), shFTO, TNC overexpression (TNC OE), and shFTO+TNC OE.. Internal control was performed with ACTB. (B) The clone formation assay was performed with AGS, BGC-823 and MGC-803 cells with control (CTRL), shFTO, TNC overexpression (TNC OE), and shFTO+TNC OE. Images shown were representative results of three biological replicates. （C） The wound healing assay was conducted using AGS, BGC-823 and MGC-803 cells with control (CTRL), shFTO, TNC overexpression (TNC OE), and shFTO+TNC OE. Images shown were representative results of three biological replicates. Scale bar: 100 μm. The data shown represented the mean ± SD. Statistical analyses were performed with the Student's t test. *$p < 0.05$; **$p < 0.01$; ***$p < 0.001$; ns stands for no significant change.
