# Supplementary material for: FTO-Driven m6A Demethylation of TNC Promotes Epithelial–Mesenchymal Transition and Gastric Cancer Metastasis
Source: Biomedicines. 2026 Aug 31;14(9):1958. doi: 10.3390/biomedicines14091958 (PMC13604668; doi:10.3390/biomedicines14091958)
Supplement: Supplementary file 1 [file biomedicines-14-01958-s001.zip › Table S1.pdf]

Table S1. Antibodies, critical commercial assays, and sequence of the primers.		
REAGENT or RESOURCE	SOURCE	IDENTIFIER
**Antibodies**		
Anti-ACTB	ABclonal	Cat# AC026
Anti-FTO	Abcam	Cat#ab124892
**Anti-YTHDF2**	Proteintech	Cat#24744-1-AP
**Anti-IGF2BP1**	Proteintech	Cat#22803-1-AP
**Anti-IGF2BP2**	Proteintech	Cat#11601-1-AP
**Anti-IGF2BP3**	Proteintech	Cat#14642-1-AP
Anti- Tenascin C	Abcam	Cat#ab108930
Anti- E-Cadherin	Cell Signaling Technology	Cat#3195
Anti- Vimentin	Cell Signaling Technology	Cat#5741
Anti-Akt	Cell Signaling Technology	Cat#9272
Anti- Phospho-Akt (Ser473)	Cell Signaling Technology	Cat# 4060S
Donkey anti-Mouse IgG-AF594	Absin	Cat# abs20017
Donkey anti-Rabbit IgG-AF488	Absin	Cat# abs20020
DAPI	Absin	Cat#abs47047616
**Critical commercial assays**		
Pierce™ BCA Protein Assay Kits	Thermo Fisher Scientific	Cat#23227
PDVF membranes	Immobilon-P	Cat# IPVH00010
Fetal bovine serum (FBS)	Thermo Fisher Scientific	Cat#A5256701
DMEM	Gibco	Cat# 11965092
RPMI 1640	Gibco	Cat# 11875093
Transwell	Corning	Cat#3422
RNA isolater reagent	Vazyme	Cat# R401-01
HiScript II Q RTSuper Mix for qPCR kit	Vazyme	Cat# R222
ChamQ SYBR qPCR Master Mix kit	Vazyme	Cat# Q311
Apoptosis Kit	BD Biosciences	Cat#559763
5-Fu	Sigma aldrich	Cat# 343922
Protease Inhibitor Cocktail	MedChemExpress	Cat# HY-K0010
Crystal violet	Beijing Solarbio Science & Technology	Cat# C8470

Dual-Luciferase® Reporter Assay System	Promega	Cat# E1910
**SC79**	MedChemExpress	Cat#HY-18749
**LY294002**	MedChemExpress	Cat#HY-10108
**actinomycin D**	**Sigma**	Cat# **A1410**
**Sequence of the primers used for RT-qPCR**		
**Gene**	**Forward Primer**	**Reverse Primer**
VIM	GACGCCATCAACACCGAGTT	CTTTGTCGTTGGTTAGCTGGT
ECAD	CGAGAGCTACACGTTCACGG	GGGTGTCGAGGGAAAAATAGG
SLUG	CGAACTGGACACACATACAGTG	CTGAGGATCTCTGGTTGTGGT
ZEB1	GATGATGAATGCGAGTCAGATGC	ACAGCAGTGTCTTGTTGTTGT
TWIST1	GTCCGCAGTCTTACGAGGAG	GCTTGAGGGTCTGAATCTTGCT
ACTB	CATGTACGTTGCTATCCAGGC	CTCCTTAATGTCACGCACGAT
FTO	ACTTGGCTCCCTTATCTGACC	TGTGCAGTGTGAGAAAGGCTT
TNC	TCCCAGTGTTCGGTGGATCT	TTGATGCGATGTGTGAAGACA
SNAI1	TCGGAAGCCTAACTACAGCGA	AGATGAGCATTGGCAGCGAG
TNC-1451	AGGGGCAGTGTGTATGTGAT	CATCATCACACTCACACCGC
TNC-2009	GGAGCAAAGATGTCCCAGTG	GCCTGTGAAGCCCTCGTG
TNC-3868	AATTTGGGAGAGGTCGTGGT	GGCTGCCTCTACTGTGTCA
TNC-4842	TCTCTGGTGCTGAACGAACT	TGTGGCACTGATGGTTTTGG
