# Supplementary material for: FTO-Driven m6A Demethylation of TNC Promotes Epithelial–Mesenchymal Transition and Gastric Cancer Metastasis
Source: Biomedicines. 2026 Aug 31;14(9):1958. doi: 10.3390/biomedicines14091958 (PMC13604668; doi:10.3390/biomedicines14091958)
Supplement: Supplementary file 1 [file biomedicines-14-01958-s001.zip › Table S2.pdf]

Table S2. Clinicopathological information for gastric cancer patients

**Characteristics**	**TNC Low (n=44)**	**TNC High (n=50)**	**P**
Age, y			0.123
<60	15	10	
>60	29	40	
Gender			0.263
Male	25	34	
Female	19	16	
Tumor differentiation			0.057
Well/Moderate	6	15	
Poor	38	35	
AJCC Stage			0.009*
I-II	23	13	
III-IV	21	37	
Venous invasion			0.249
No	33	32	
Yes	11	18	
Nervous invasion			0.367
No	39	41	
Yes	5	9	

AJCC American Joint Committee on Cancer, 8th edition. *$P < 0.05$.
